# Root and Root Canal Configuration Characterization Using Microcomputed Tomography: A Systematic Review

**DOI:** 10.3390/jcm11092287

**Published:** 2022-04-20

**Authors:** Mohmed Isaqali Karobari, Sohaib Arshad, Tahir Yusuf Noorani, Naveed Ahmed, Syed Nahid Basheer, Syed Wali Peeran, Anand Marya, Charu Mohan Marya, Pietro Messina, Giuseppe Alessandro Scardina

**Affiliations:** 1 Conservative Dentistry Unit, School of Dental Sciences, Health Campus, Universiti Sains Malaysia, Kubang Kerian, Kota Bharu 16150, Kelantan, Malaysia; 2 Department of Conservative Dentistry & Endodontics, Saveetha Dental College & Hospitals, Saveetha Institute of Medical and Technical Sciences University, Chennai 600077, Tamil Nadu, India; 3 Periodontics Unit, School of Dental Sciences, Health Campus, Universiti Sains Malaysia, Kubang Kerian, Kota Bharu 16150, Kelantan, Malaysia; ehab_arshad@hotmail.com; 4 Department of Medical Microbiology and Parasitology, School of Medical Sciences, Universiti Sains Malaysia, Kubang Kerian, Kota Bharu 16150, Kelantan, Malaysia; naveed.malik@student.usm.my; 5 Department of Restorative Dental Sciences, College of Dentistry, Jazan University, Jazan 45142, Saudi Arabia; snbasheer@jazanu.edu.sa; 6 Department of Periodontics, Armed Forces Hospital Jizan, Jazan 82722, Saudi Arabia; doctorsyedwali@yahoo.com; 7 Department of Orthodontics, Saveetha Dental College & Hospitals, Saveetha Institute of Medical and Technical Sciences University, Chennai 600077, Tamil Nadu, India; amarya@puthisastra.edu.kh; 8 Department of Public Health Dentistry, Sudha Rustagi College of Dental Sciences and Research, Faridabad 121002, Haryana, India; maryacm@yahoo.co.uk; 9 Department of Surgical, Oncological and Stomatological Disciplines, University of Palermo, 90133 Palermo, Italy; pietro.messina01@unipa.it

**Keywords:** dental anatomy, dental pulp, dental diagnostic imaging, endodontics, morphology, Micro-CT, root, root canal

## Abstract

This systematic review’s objective was to conduct a complete analysis of the literature on the root canal morphology using advanced micro-computed tomography. The electronic web databases PubMed, Scopus, and Cochrane were examined for research papers concerning the chosen keywords, evaluating the root canal morphology using Micro-CT, published up to 2021. The articles were searched using MeSH keywords and searched digitally on four specialty journal websites. DARE2 extended (Database of Attributes of Reviews of Effects) was used to assess bias risk. The information was gathered from 18 published studies that strictly met the criteria for inclusion. In the included studies, a total of 6696 samples were studied. The studies were conducted on either maxillary (*n*-2222) or mandibular teeth (*n*-3760), permanent anteriors (*n*-625), and Third molars (*n*-89). To scan samples, a Scanco Medical machine in was used in 10 studies, Bruker Micro-CT in 34, and seven other machines were utilized in the rest. Bruker Micro-CT software from Kontich, Belgium, VG-Studio Max 2.2 software from Volume Graphics, Heidelberg, Germany, was the most commonly used software. The minimum Voxel size (resolution) adopted in the included studies was 11.6 µm. However, 60 µm was the maximum. Most studies classified the root canal morphology using Vertucci’s classification system (*n*-16) and the four-digit system (*n*-6).

## 1. Introduction

Endodontic therapy aims to thoroughly clean and obturate the whole system of the root canal. However, to prevent endodontic failure and execute successful root canal therapy, specific determinants play an essential role [1,2]. Precise shaping, cleaning, and filling of all spaces previously filled by the radicular pulp tissues or pulp capping/pulpotomy to maintain healthy dental pulp is required. The morphologic uniqueness of each root necessitates a comprehensive knowledge of variations in the root canal system, which should be reflected during the diagnostic and treatment process [3]. Research upon the root canal morphology in lasting teeth has revealed that the root canal’s amount and classification can differ by ethnicity, gender, and in different populations, within the same population, and uniquely in each person [4,5]. Furthermore, it is possible that a variety of morphologic root canal system configurations exist; as a result, each tooth should be evaluated separately using a proper classification system [6].

The investigation of external and internal anatomy of different teeth using many in vitro and in vivo techniques were performed in the beginning of 20th century [7]. Various in vitro techniques were used to identify root and canal morphology which includes root sectioning, staining, tooth clearing, microscopic investigation, radiographic investigations using conventional radiographs, and three-dimensional techniques such as CBCT and microcomputed tomography (MCT) [8]. The in vivo techniques include conventional radiographic examinations, retrospective evaluation of patients’ data, clinical evaluation during root canal treatment, digital radiography, and advanced radiographic techniques such as CBCT [8]. An investigation showed a technique by using longitudinal sectioning to produce a sagittal view of pulp space from pulp chamber to the root apex [9]. Opaque wax was used to fill the exposed canals, but this method showed lateral canals very rarely [10]. Rosenstiel (1957) introduced a technique using a Radio opaque material to reproduce the root canals. A study demonstrated a simple in vitro technique to evaluate both endodontically treated and untreated root canal systems. The following steps were used in the technique to make teeth transparent. Firstly, teeth were decalcified using nitric acid, then dehydrated using alcohol, and finally cleared with methyl salicylate [11].

**Figure 1:**
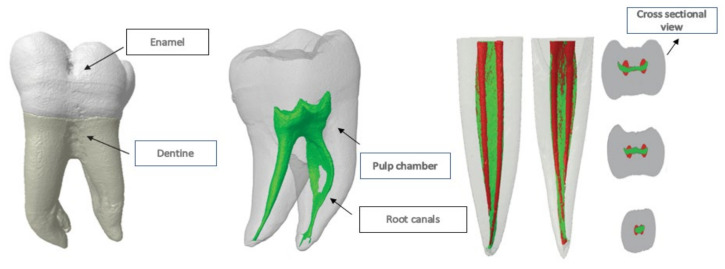
Micro-CT in endodontics.

Digital radiography, magnetic resonance imaging (MRI), densitometry, ultrasound, and computed tomography (CT) are just a few of the noninvasive dental imaging technologies that have been developed in recent years. However, most of these approaches are restricted since they only provide a 2-dimensional (2-D) examination of the root canal system (RCS) and cannot be easily compared subjectively or quantitatively with other samples [12]. Furthermore, these methods do not allow for synchronized 3-dimensional (3-D) examination of teeth surface and interior anatomy [13]. In endodontic research, microcomputed tomography (Micro-CT) has acquired much interest since it displays high-resolution (10 µm) tooth morphological structures and has proven to be an essential information source for dentists, as shown in Figure 1 [14]. Microcomputed tomography is a nondestructive and reproducible ex vivo research method and is considered as the research method that offers the foremost possibility for an accurate examination of the morphology of the root canal system.

The ex vivo investigation is used in Micro-CT imaging, which is widely accepted because of its accuracy, repeatability, and noninvasiveness, all of which are superior to other commonly used research methods [15]. Maxillary central incisors have one root and one main canal. Rarely, at a 6% rate, one canal of central maxillary teeth splits into two parts at the apical foramina which can be classified as Vertucci type V [16]. Apical root canal morphology should be considered because of its main effect on the success of root canal treatment. In the study of Adorno et al. [17], accessory canals in the apical 3 mm in the Japanese population were found among 46% of the specimens. Over the years, different studies have been conducted to understand the root canal morphology of premolars using different research methods [18] and different populations [19,20,21]. The frequency of a single canal is 54–88.5%. However, multiple canals were reported in 11.5–46% of cases [21,22]. In the study of Pan et al. [23], the prevalence of maxillary first premolar teeth with one main root canal was 67.8%, with two roots at 31.9%, and with two canals at 88.2%. In the Malaysian population, according to Vertucci’s classification, the second premolar was detected as single-root type I with the rate of 58.2% [16]. In posterior teeth, mandibular first molars are recognized to exhibit various complex and distinct morphological variations of the root canal system [24,25]. This tooth usually has two roots, but sometimes it has three, with two or three canals in the mesial root and one, two, or three canals in the distal root [26,27]. When only one distal root canal is present, it is often buccolingually oval, and untreated surface areas were shown to be as high as 59–79% when rotary instruments were used for the shaping procedure [28].

Similarly, a study on the Burmese population showed that the prevalence of two canals in mesiobuccal roots of the upper first molar teeth decrease gradually towards the upper third molars. About 85.2% of the 270 roots of the maxillary teeth had one root canal at the apex, 14% had two apical canals, and 0.8% had three apical canals [25,29]. Moreover, the morphology of root canals was explored, white spot lesions on enamel were identified, and enamel demineralization with therapy were assessed using Micro-CT [30,31]. The latest evidence demonstrated the ability to scan isthmuses successfully, while another claimed to detect inorganic material within a tooth root [32,33]. The nondestructive Micro-CT imaging method allows for multiple exposures and data collection. As a result, this imaging method is beneficial for evaluating experimental endodontics [34]. The goal of this systematic review was to carry out a thorough examination of the literature on root canal physiology using sophisticated microcomputed tomography.

## 2. Methods

### 2.1. Study Protocol and Registration

The Reporting Items preferred for Meta-Analysis and systematic Review (PRISMA) procedures (http://www.prisma-statement.org, accessed on 10 April 2022) were respected in this work. The current systematic review is registered in PROSPERO with the number CRD42021278968.

### 2.2. Research Question

Studies about assessment of root canal morphologies through microcomputed tomography were selected based on the “PICOS” (PRISMA-P 2016) technique:

- P (population): Extracted teeth models
- I (intervention): Assessment by Micro-CT
- C (comparison): None
- O (result): Root and root canal morphologies
- S (study design): In vitro studies

### 2.3. Search Strategies

The electronic online databases search was conducted for research papers based on selected keywords, assessing root canal morphology using Micro-CT, published until March 2022. The number of studies obtained from each dataset is displayed in Table 1. The articles were searched using MeSH keywords and searched digitally on four specialty journal websites. The MeSH keywords were searched in PubMed and Scopus initially, as per our initial search criteria. Further, to add more scientific evidence related to the topic, the search was carried out in Cochrane. Furthermore, to include the latest articles up to the last search date published in the specialty journals as it may take some time for the articles to be included in the indexes after they are published, the search on websites of four endodontic specialty Journals were performed, including the Journal of Endodontics, the International Endodontic Journal, the Australian Endodontic Journal, and the Iranian Endodontic Journal.

### 2.4. Data Sources

Two separate researchers (M.I.K and N.A.) performed an electronic literature search on 20 March 2022, using MeSH terms and keywords, as well as the Boolean operators “OR” and “AND” to compile relevant material using appropriate filters. The keywords used were “Tooth Root/anatomy and histology”, “Tooth Root/diagnosis”, “Tooth Root/diagnostic imaging”, “Tooth root”, “dental pulp cavity”, “Micro-CT”, and “X-ray Microtomography/methods”. The required literature was then gathered using proper filters by combining these key terms with the Boolean operators “OR” and “AND” as shown in Table 1. Furthermore, a hand search was also conducted by two different reviewers using keywords such as “Root canal morphology,” “Root canal configuration,” “Root canal system,” “Microcomputed tomography,” “Micro-computed tomography,” and “Micro-CT” from databases such as PubMed, Scopus, ScienceDirect, and Cochrane.

### 2.5. Eligibility Criteria

**Figure 2:**
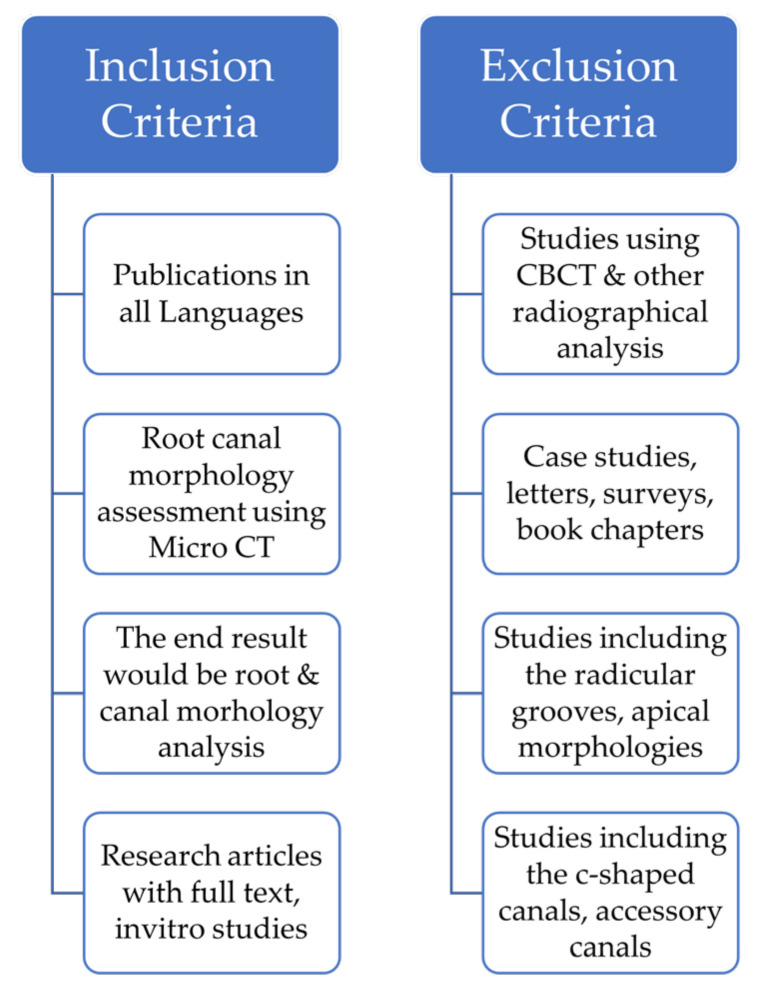
Inclusion and Exclusion Criteria.

A literature search was performed to uncover studies that used Micro-CT to assess root canal morphology. Two reviewers used the PICOS approach to examine the entire texts of the remaining papers and set inclusion and exclusion criteria. The year of publication was not restricted in any way. On 20 March 2021, the final database search was accomplished. A third reviewer’s decision was used to settle disagreements. Figure 2 illustrates the inclusion and exclusion criteria.

### 2.6. Study Selection

The studies that examined the assessment of root canal morphology using the Micro-CT technique, which are published in various medical journals, were found through a random check of research papers from online sources. Two researchers evaluated relevant studies against previously defined inclusion and exclusion criteria to substantiate the search technique, as shown in Figure 2.

### 2.7. Data Extraction

Two reviewers (M.I.K and S.A.) assessed the titles and abstracts of the publications for the inclusion/exclusion criteria mentioned above, and “relevant” articles were chosen for a full-text reading. This procedure was carried out independently, with the help of a third researcher (NA), in the event of any questions or conflicts. A manual hand search was also carried out using different keywords, and studies were included based on selected criteria.

### 2.8. Quality Assessment and Risk of Bias of Research Articles

The papers were selected for inclusion and exclusion based on their titles, abstracts, and inclusion and exclusion criteria. Following the screening process, full-text articles were reviewed one by one, and the material’s quality was evaluated. The articles were rated for allotment biases, preference biases, involvement integrity, allocation concealment, withdrawals and dropouts, confusion, data collection methods, and statistical analysis using internal and external validity guidelines. A total of 60 papers were screened for quality, with nine being rejected due to a lack of information about the processes, teeth, research nature, and outcomes.

The Joanna Briggs Institute (JBI) critical assessment checklist was used to appraise the quality of the included studies [35]. This checklist assessed nine items: (i) appropriate sampling frame, (ii) proper sampling technique, (iii) adequate sample size, (iv) study subject and setting description, (v) sufficient data analysis, (vi) use of valid methods for the identified conditions, (vii) valid measurement for all the participants, (viii) use of appropriate statistical analysis, and (ix) adequate response rate. Answers such as yes, no, unclear, or not applicable are assigned to each item. The ‘yes’ response received a 1 score, whereas the ‘no’ and ‘unclear’ responses received ratings of 0. Finally, the average score for each item was computed. The quality of studies with scores below and above the mean was then classified as good or poor quality, respectively. The study was included or excluded based on the methodological quality assessment. (Supplementary Table S1).

Two researchers (M.I.K. and N.A.) oversaw scoring, and they used the JBI criteria to base their scores. After comparing the results of their individual questions, they resolved any discrepancies to arrive at an ‘agreed score’. All of the 51 included studies individually had a total score of ≥70%. Hence, both the researchers (M.I.K and N.A.) showed agreement for most of the included studies and were given >70% scores, thus limiting the bias.

## 3. Results

### 3.1. Study Selection Results

**Figure 3:**
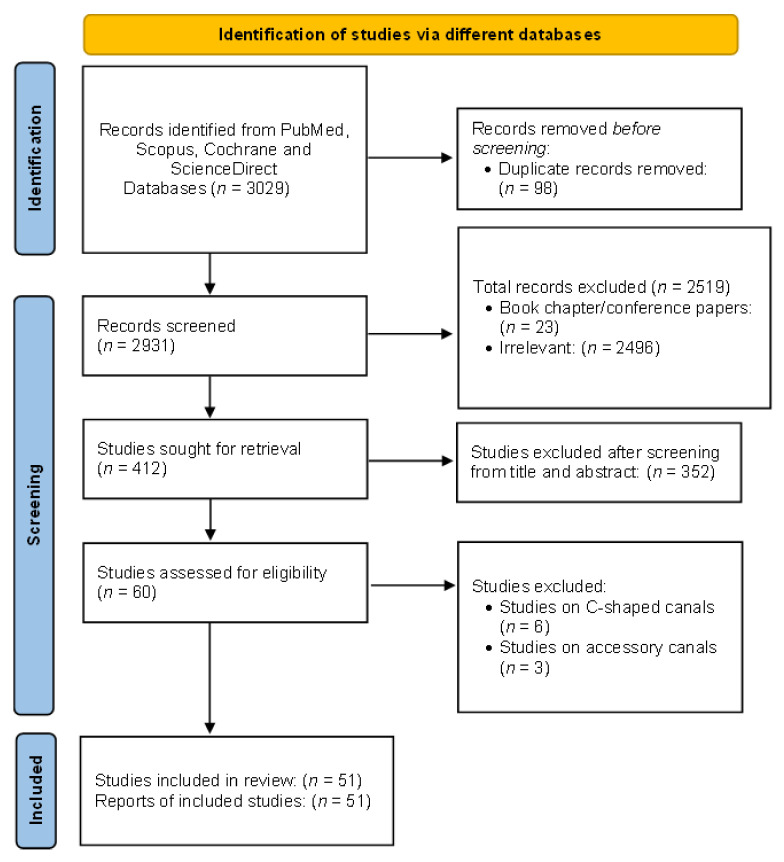
PRISMA flowchart showing the selection process of articles retrieved from different web sources.

PubMed yielded a total of 236 research papers using MeSH keywords, 483 from Cochrane using MeSH keywords, 131 from Scopus using MeSH keywords, and 2179 from ScienceDirect. Following the removal of duplicate articles (98), a total of 2931 studies were recognized for further consideration. After reading the titles of the articles, another 2519 were eliminated. In addition, 352 additional articles were eliminated after reading the abstracts. By reading the full texts of the residual 60 articles, they were evaluated for further selection; nine more articles were eliminated. The data were extracted from the 51 studies that strictly met the eligibility criteria. Figure 3 depicts the selection criteria as it follows the PRISMA guidelines. These 51 articles were examined for the current study based on the quality of the research studies.

### 3.2. Study Features

The studies’ basic features included in the systematic review are summarized in Table 2. The studies were performed in various countries, lasted varying amounts of time, and were published in various journals. Each included study was published in a good, reputed journal indexed in Web of Science/PubMed/Scopus. The technical characteristics, such as sample size, type of teeth, instrument used, resolution, software, classification system, methods, outcomes, and conclusion, were omitted from the systematic review. Most of the reports comprised were issued in the Journal of Endodontics (*n*-13). In contrast, six were published in the International Endodontic Journal, four in the Clinical Oral Investigations, two in the Scientific reports, two in the Journal of Conservative Dentistry, two in Oral Surgery, Oral Medicine, Oral Pathology, Oral Radiology, and Endodontology, two in the Archives of Oral Biology, two in the Journal of Applied Oral Sciences, two in Clinical Oral Investigations, two in Clinical Anatomy, one in the Australian Endodontic Journal, one in the International Journal of Oral Sciences, one in the Swiss Dental Journal, one in the European Endodontic Journal, one in Acta Odontologica Latinoamericana, one in the Journal of Dental Sciences, one in the Nigerian Journal of Clinical Practice, one in the International Medical Journal of Experimental and Clinical Research, one in the British Journal of Oral and Maxillofacial Surgery, one in The Saudi Dental Journal, one in Imaging Science in Dentistry, one in Medical Principles and Practice, one in The Bulletin of Tokyo Dental College, and one in Annals of Anatomy. All the included studies were published between 2008–2022. The research included was carried out in a variety of nations, including Brazil (*n*-16), China (*n*-7), Egypt (*n*-5), Germany (*n*-5), Poland (*n*-3), the United States (*n*-2), Korea (*n*-2), Turkey (*n*-2), New Zealand (*n*-1), Chile (*n*-1), France (*n*-1), Myanmar (*n*-1), Saudi Arabia (*n*-1), Italy (*n*-1), Japan (*n*-1), and the United Arab Emirates (*n*-1)

In the studies included, a total of 6696 samples were studied. The studies were conducted on either maxillary (*n*-2222) or mandibular teeth (*n*-3760), permanent anteriors (*n*-625), and third molars (*n*-89). Of the total maxillary and mandibular teeth, 970 were maxillary first molars, 262 were maxillary second molars, 659 were both maxillary first and second molars, 331 were maxillary premolars, 789 were mandibular first molars, 158 were mandibular second molars, 529 were mandibular first premolars, 1254 were mandibular incisors, 281 were mandibular canines, and the remaining 1463 were mixed. The authors used different reagents to store the samples (70% Alcohol, 0.5% sodium azide solution, or 10% formalin). To scan samples, a Scanco Medical machine was used in 10 studies, a Bruker Micro-CT machine in 34 studies, Micro-CT Inveon, Siemens Medical Solutions, Knoxville in two studies, VGStudio Max 2.2 in one study, Nikon Metrology Inc, Brighton in one study, Nanotom S, General Electric in one study, Kodak, Rochester, New York, USA in one study, and HMX 225-ACTIS 4, Tesco, Inc in one study.

Bruker Micro-CT software from Kontich, Belgium (*n*-27), software VG-Studio Max 2.2 from Volume Graphics, Germany Heidelberg (*n*-10), NRecon software (*n*-5), CTAn v.1.12 software Mimics 17.01, Materialize, Leuven, Belgium (*n*-4), MICs 10.01 software Materialise, Leuven, Belgium (*n*-1), Image processing language (*n*-1), On-Demand 3D software from Cybermed, Seoul, Republic of Korea (*n*-2), Cobra software Siemens Medical Solutions, Knoxville (*n*-1), and MeVisLab v3.2 software (MeVis Medical Solutions AG, Bremen, Germany) (*n*-1) was used in the studies to interpret the data about root canal morphologies. The minimum Voxel size (resolution) adopted in included studies was 11.6 µm. However, 60 µm was the maximum resolution adopted in included studies. Most studies classified the root canal morphology using Vertucci’s classification system (*n*-16) and the four-digit system (*n*-6). Furthermore, Weine’s classification system (*n*-3), Pucci & Reig (1944), a new classification system by Ahmed et al., Pomeranz ‘s classification and the American Association of Endodontics system for classification were also used. The technical characteristics of the studies are shown in Table 3.

## 4. Discussion

Micro-CT analysis has proven useful in a wide variety of applications in dental research. It can provide high-resolution images, as well as qualitative and quantitative analysis of teeth [83,84]. To achieve long-term treatment success, endodontic anatomical knowledge is required. As a result, a detailed description of the apical region is required [85]. Until now, there was a scarcity of detailed information on the anatomy of the RCS; therefore, 3-D, high-resolution techniques dominated. Compact commercial systems are now available and are quickly becoming vital in many academic and corporate research laboratories. It is possible to study a wide range of specimens using Micro-CT to examine mineralized tissue, teeth, bone, and materials such as ceramics, polymers, and biomaterial scaffolds [86,87,88,89]. Micro-CT provides a repeatable, nondestructive, and noninvasive technique for nonclinical ex vivo evaluation with this goal in mind, enabling measured values of the structures investigated and providing critical info regarding minimal structures such as the end part of the apical portion of teeth [49,50,90,91].

Even though data is challenging to come by, it appears that a large group of researchers agree that Micro-CT gives more objective information than traditional 2-D optical techniques [92], the clearing procedure, or scanning microscopy [44]. As a result, in the present study, a substantial number of sufficiently recognized teeth were evaluated using Micro-CT, allowing for a thorough statistical analysis of the sample. Compared with other investigating techniques, the advantages of Micro-CT produce extraordinary resolution 3-D and 2-D figures, with possibilities of rescanning the sample and volumetric analysis of external and internal structures. The Micro-CT system using a microfocal spot X-ray source and a high-resolution detector is projected in several directions to obtain a three-dimensional reconstructed image of the sample. Since the imaging process is nondestructive, the unique properties of the same sample can be tested multiple times, and the sample can still be used after scanning for further biological and mechanical testing [89]. Some of the recent applications of Micro-CT in dental research includes enamel thickness and tooth measurement [93], analysis of root canal morphology and evaluation of root canal preparation [94], craniofacial skeletal development and structure [95], biomechanics, tissue engineering, determination of mineral concentrations of teeth [96], and the measurement of implant stability and osseointegration [97]. The main disadvantage of Micro-CT is that it cannot be utilized in medical practices due to elevated radiation heights, the operating cost, time taken to process data, cost-effectiveness, and safety [98,99].

The current study provides an overview of the Micro-CT studies for root canal morphology. The data included in this systematic review are secondary information collected from various past research studies. Secondary data are prone to flaws or biases present in the original data, which might eventually appear in the study’s findings. For example, it may appear in the analysis technique, or the smallest number of teeth examined in the research. However, the goal was to give the dentistry and endodontic communities a Micro-CT-based analysis of the massive data on the root canal morphology.

Endodontic therapy, both nonsurgical and surgical, needs a thorough understanding of tooth anatomy and morphology [100,101]. Because it is used to instrument and fill root canals to a considerable extent, the morphological interpretation of the apical region should be accurate. Understanding the apical region and the configuration of the root canals is an essential and challenging condition that the clinician must have to make judgments about during endodontic therapy [102].

Despite the fact that the permanent anterior maxillary and mandibular teeth are typically single-rooted, studies suggest that an auxiliary root might be present [103]. Earlier studies in other ethnicities, comprising Turkish, American, Brazilian, and Indian communities, found that all maxillary incisor teeth were single-rooted [104,105,106]. This suggests that the number of roots in maxillary incisors does not differ structurally throughout all populations. Nevertheless, it is important to note that the presence of a double-rooted maxillary anterior has only been confirmed in a few case studies [103]. However, studies revealed a double-rooted mandibular anterior [4]. Numerous root canal morphology differences in mandibular incisors were documented [37,43,47]. The current review showed that the most common type of root canal morphology classified using the four-digit system was 1-1-1, Type I using Vertucci’s classification system, and Type 1-1 using Weine’s classification. According to previous research, Vertucci type I in mandibular incisors can range between 55% and 87% [107]. In two earlier investigations of Turkish populations, type I in the mandibular incisors was lower [108,109]. The most common type of root canal configuration identified in this study was type I (75 percent), similar to Vertucci’s results [16]. Both mandibular incisors had 88% of type 1 configuration, according to Madeira and Hetem [110]. Furthermore, De Almeida, MM et al. revealed that the most common was type III from the Vertucci classification (16%) [47].

The mandibular first molar is not only the most treated endodontically, but it also presents several anatomic difficulties. The diversity includes isthmuses, several canals, apical ramifications, and lateral canals [3]. Additionally, the distal surface of the mesial root has a thin patch of dentin referred to as a danger zone because there is a higher chance of perforation of dentin in this region during mechanical instrumentation. Hence, orthograde and retrograde endodontic treatment in this tooth may be difficult because of its unusual form [40,102]. The anatomy of the mandibular second molar has been widely investigated, notably using the cleaning procedure [102]. According to previous research, the frequency of C-shaped canals is between 31 and 45% (mostly of Asian people) [52,111,112,113]. Various classifications of the tridimensional distributions of RCS and the transverse sections have also been published subsequently [114,115].

The canal shape of posterior maxillary teeth varies significantly between races and geographical locations. As per Mohara et al., Brazilians have a 64.2 percent frequency of MB2 in the foremost permanent molar and a 33.5 percent incidence in the subsequent permanent maxillary molar, respectively [116]. In South Africa, type IV root canals are widespread in maxillary primary molar and type I root canals are the most familiar in a maxillary second molar, according to the Vertucci classification of the root canal [117]. According to Li et al., the most common maxillary first premolar anatomy in the Chinese population is one root with two canals (58.0 percent), and the most common canal morphology is type IV (42.7 percent) [118]. Guo et al., on the other hand, examined the maxillary first molars’ morphology amongst the North American population and discovered that Asians had a higher occurrence of type I (35.0 percent) and type IV (45.0 percent) configurations than whites (type IV: 36.3 percent, type I: 23.4 percent) [119]. As a result, root canal shape varies depending on where you live. Relevant studies in native communities can help dentists better analyze and understand root canal therapy while also adding to the body of information about root and canal morphology in humans.

The current work uses Micro-CT imaging of many samples to provide a detailed assessment of root canal morphology of the mandibular and maxillary teeth. This knowledge will help practitioners comprehend and anticipate the challenges of 3-D endodontic therapy, particularly during root canal shaping and cleaning. This study revealed that the maxillary first molar and mandibular first premolars had a higher incidence of morphological endodontic variables than the other maxillary and mandibular molars and incisors, indicating that they are more complicated.

## 5. Study Limitations

We searched data from a small number of significant websites for our systematic review. Articles that have appeared in other publications not indexed in the indices searched may have been ignored. We have also only included items published in English; as a result, publications in other languages may have been overlooked. A limited number of studies have been performed using Micro-CT.

## 6. Conclusions

This review used Micro-CT studies to provide detailed information atop the anatomy of the root and canal morphology in permanent dentition of various populations. Further, it revealed wide disparities concerning root and canal morphology in permanent dentition, which could perhaps derive from the geographical area studied. In Micro-CT findings, the mandibular incisors followed by maxillary molars were the most studied teeth. The use of multiple categorization systems and Micro-CT allowed for a more precise description of the root canal system and its ramifications, with some inconsistencies noted for molars. This Micro-CT study adds to the existing categorization methods by providing a detailed description of the diversity among root canals and their ramifications and clinically relevant data on the presence and location of lateral canals in all human tooth groups.

## Figures and Tables

**Table 1:** Information of sources and search strategies using MeSH keywords.

Database	Search Strategies	Results
PubMed	((((((((((((Tooth Root[Title/Abstract]) OR (Tooth anatomy[Title/Abstract])) OR (Tooth histology[Title/Abstract])) OR (Tooth diagnosis[Title/Abstract])) OR (Tooth diagnostic imaging[Title/Abstract])) OR (Root canal morphology[Title/Abstract])) OR (Root canal configuration[Title/Abstract])) OR (Root canal system[Title/Abstract])) OR (Dental Pulp Cavity[Title/Abstract])) OR (Dental anatomy[Title/Abstract])) OR (Dental histology[Title/Abstract])) OR (Dental diagnosis[Title/Abstract])) OR (Dental diagnostic imaging[Title/Abstract]) **AND** (((((((X-ray Microtomography[Title/Abstract]) OR (X-ray methods[Title/Abstract])) OR (Micro-CT[Title/Abstract])) OR (micro computed tomography[Title/Abstract])) OR (microcomputed tomography[Title/Abstract])) OR (microcomputed tomography[Title/Abstract])) OR (Micro-CT[Title/Abstract])) OR (Micro-CT[Title/Abstract])	236
Scopus	Tooth Root OR Tooth anatomy OR Tooth histology OR Tooth diagnosis OR Tooth diagnostic imaging OR Root canal morphology OR Root canal configuration OR Root canal system OR Dental Pulp Cavity OR Dental anatomy OR Dental histology OR Dental diagnosis OR Dental diagnostic imaging **AND** X-ray Microtomography OR X-ray methods OR Micro-CT OR micro computed tomography OR microcomputed tomography OR microcomputed tomography OR Micro-CT OR Micro-CT	131
Cochrane	Tooth Root OR Tooth anatomy OR Tooth histology OR Tooth diagnosis OR Tooth diagnostic imaging OR Root canal morphology OR Root canal configuration OR Root canal system OR Dental Pulp Cavity OR Dental anatomy OR Dental histology OR Dental diagnosis OR Dental diagnostic imaging **AND** X-ray Microtomography OR X-ray methods OR Micro-CT OR micro computed tomography OR microcomputed tomography OR microcomputed tomography OR Micro-CT OR Micro-CT	483
ScienceDirect	(Root canal morphology OR Root canal configuration OR Root canal system OR Dental Pulp Cavity) AND (Micro-CT OR micro computed tomography OR microcomputed tomography OR Micro-CT OR Microtomography)	2179
Total		3029

**Table 2:** Studies included in the systematic review.

No.	Study Reference	Journal	Population	Year of Publication
1	[36]	Journal of Endodontics	Egyptians	2015
2	[37]	Journal of Endodontics	Germans	2019
3	[3]	Journal of Endodontics	Swiss Germans	2020
4	[38]	Journal of Applied Oral Sciences	Brazilians	2019
5	[39]	Journal of Endodontics	Americans	2013
6	[40]	International Endodontic Journal	Brazilians	2015
7	[41]	Clinical Oral Investigations	Koreans	2012
8	[42]	Australian Endodontic Journal	Brazilians	2018
9	[43]	Journal of Endodontics	Brazilians	2014
10	[44]	Journal of Endodontics	Brazilians	2015
11	[45]	Journal of Endodontics	Brazilians	2016
12	[46]	International Endodontic Journal	New Zealanders	2011
13	[47]	Journal of Endodontics	Brazilians	2013
14	[48]	International Endodontic Journal	Brazilians	2017
15	[49]	Journal of Endodontics	Egyptians	2016
16	[50]	International Journal of Oral science	Egyptians	2017
17	[51]	Journal of Endodontics	Swiss Germans	2020
18	[52]	Archives of Oral Biology	Chinese	2018
19	[53]	Journal of Conservative Dentistry	Brazilians	2018
20	[54]	Scientific Reports	Chilean	2021
21	[55]	Journal of Conservative Dentistry	Brazilians	2018
22	[56]	Swiss Dental Journal	Egyptians	2017
23	[57]	Journal of Endodontics	Americans	2019
24	[58]	European Endodontic Journal	Brazilians	2020
25	[59]	Clinical Anatomy	Polandians	2018
26	[60]	Acta Odontológica Latinoamericana	Brazilians	2020
27	[61]	Journal of Dental Sciences	Chinese	2022
28	[62]	Oral Surgery, Oral Medicine, Oral Pathology, Oral Radiology, and Endodontology	Italians	2008
29	[63]	Journal of Endodontics	Brazilians	2013
30	[64]	Clinical Oral Investigations	Chinese	2021
31	[65]	Scientific Reports	Chinese	2017
32	[66]	Nigerian Journal of Clinical Practice	Egyptians	2020
33	[67]	International Medical Journal of Experimental and Clinical Research	Chinese	2021
34	[68]	British Journal of Oral and Maxillofacial Surgery	France	2005
35	[1]	International Endodontic Journal	Brazilians	2013
36	[69]	Clinical Oral Investigations	Chinese	2021
37	[70]	International Journal of Dentistry	Myanmar	2021
38	[71]	The Saudi Dental Journal	Saudis	2016
39	[72]	International Endodontic Journal	Brazilians	2012
40	[73]	Scientific Reports	Swiss Germans	2021
41	[74]	Clinical Oral Investigations	Chinese	2013
42	[75]	Journal of Applied Oral Science	Brazilians	2016
43	[76]	Journal of Endodontics	Swiss Germans	2020
44	[77]	Imaging Science in Dentistry	Turkish	2021
45	[78]	International Endodontic Journal	Italians	2009
46	[79]	Clinical Anatomy	Polish	2018
47	[22]	Medical Principles and Practice	Emiratis	2017
48	[80]	The Bulletin of Tokyo Dental College	Japanese	2011
49	[81]	Oral Surgery, Oral Medicine, Oral Pathology, Oral Radiology, and Endodontology	Koreans	2009
50	[12]	Annals of Anatomy	Polish	2018
51	[82]	Archives of Oral Biology	Turkish	2020

**Table 3:** Characteristics of the included studies.

Study Reference	Sample Size	Sample Type	Micro-CT Machine	Voxel Size (Resolution)	Software Used	Classification System	Technique	Results	Conclusion
[36]	179	Maxillary first molars *	Scanco Medical, Bruttisellen, Switzerland	20 µm	Heidelberg, Germany Volume Graphics, VG-Studio Max 2.2;	fourfour-digit system	Endodontic access cavity was prepared with a high-speed and round bur, pulp stones were removed by ultrasonic scaler if needed, and at the end, the pulp chamber was rinsed with sodium hypochlorite following suction for drying and assessment of Micro-CT morphology of the root canal.	In mesiobuccal roots, the most prevalent root canal configurations were 1-1-1/1 (45.8%), 1-1-2/2 (25.1%), and 1-2-2/2 (10.1%), while in distobuccal roots, the most prevalent root canal configurations were 1-1-1/1 (97.2%) and 1-1-1/1 palatal (10.1%) (98.9 percent).	Maxillary first molars have a wide range of root canal configurations. According to this study, the mesiobuccal root has only one main foramen and one root canal entrance.
[37]	125	Permanent Anteriors	Scanco Medical, Bruttisellen, Switzerland	20 µm	Heidelberg, Germany Volume Graphics VG-Studio Max 2.2;	four-digit system	Rendering software was used to visualize the various tooth structures generated from the 3-D reconstructions of the Micro-CT scans. Red was used to color the pulp chamber and RCS, white for the enamel/crown area, and transparent grey for the root/dentin area. The root canal configuration was determined when the roots were split into three.	The most prominent root canal configurations were 1-1-1/1 (56%), 1-2-1/1 (17.6%), and 1-1-1/2 (10.4%); however, 19 teeth had a total of nine root canal combinations (15.2 percent).	The study discovered variations in the morphology of root canal of anterior teeth in the German population, the most prevalent root canal configuration being 1-1-1/1.
[3]	109	Mandibular first premolar *	Scanco Medical, Bruttisellen, Switzerland	16 µm	Heidelberg, Germany Volume Graphics VG-Studio Max 2.2;	four-digit system	The pulp chamber and root canals were red, the enamel was white, and the dentin was a translucent grey tint to distinguish tooth features. The root canal configuration was determined by dividing the roots into thirds and using the RCC method to generate a four-digit code system. The first three digits of the code system indicate the number of root canals at the coronal boundary of the coronal, middle, and apical thirds of a root; the fourth digit, divided by a slash, represents the number of physiological foramina.	The most common root canal configurations were 1-1-1/1 (70.6%), 1-1-2/2 (7.3%), 1-2-2/2 (7.3%), and 1-2-1/1 (7.3%). (5.5%).	The researchers discovered differences in the morphology of root canal of maxillary first premolars in the German-Swiss population, with 1-1-1/1 root canal configurations being the most prevalent (70.6 percent).
[38]	500	Maxillary and mandibular: Anteriors, premolars, and molars	Bruker Micro-CT, Kontich, Belgium	26 μm	Bruker Micro-CT, Kontich, Belgium	Pucci & Reig (PR) (1944) and American Association of Endodontics	CT scanning for root canal morphology.	According to the PR, significant canals were found in 100% of the teeth studied, with the exception of the second mesiobuccal canal in the maxillary first and second molars, which had a frequency of 87 and 75 percent, respectively. In terms of the major canal, the AAE classification revealed the same results as the PR classification.	The variation of the RCS was accurately described by Micro-CT, which was proved by the PR and AAE classifications, with some discrepancies observed for upper molars.
[39]	18	human hemi-maxilla	Scanco Medical, Bruttisellen, Switzerland	20 µm	Image processing language (version 5.15; Scanco Medical)	--	The hemi-maxillae were taken from cadavers used in medical research and teaching (with prior agreement). Teeth from the human hemi-maxillae were extracted, washed with 3% NaOCl, and imaged using a CT scanner.	Thirteen first molars and fourteen second molars from eighteen cadavers were studied. Two canals were found in 100% of maxillary first molar MB roots (100 percent). Two canals were found in 57 percent of maxillary second molar MB roots.	Micro-CT canal numbers were considerably different from digital periapical radiograph counts in cadaver maxillary teeth, but not from 3-D CBCT counts.
[40]	32	Mesial roots of mandibular first molars	Bruker Micro-CT, Kontich, Belgium	19.6 µm	Bruker Micro-CT, Kontich, Belgium	Vertucci’s classification	Group 1 and Group 2 (*n* = 10), Group 3 (*n* = 12). Based on Micro-CT scans and presenting several canal configurations, were evaluated followed by a clearing technique.	Type I root canals were found in a considerably limited number of cleaned teeth, but type II root canals were found in all cases.	The evaluation technique and anatomy type significantly affected the accuracy of detecting mesial root canal shape among the tooth population examined.
[41]	154	Extracted human maxillary first molar mesiobuccal roots **	SkyScan, Aartselaar, Belgium	15.9 µm	On-Demand 3D software (Cybermed, Seoul, Republic of Korea).	Vertucci’s classification	The mesiobuccal roots of maxillary first molars with more than two canals were examined using 154 Micro-CT scans. Weine and Vertucci’s classifications were used to classify the root canal configurations of multiple-canalled MB roots.	73.4 percent of the MB roots had multiple canals. The most common canal type was Weine type III. In 29.2 percent and 17.7 percent of MB roots, respectively, nonclassifiable configuration types were found.	The current study indicates that configuration classifications may need to be modified to reflect MB root morphology better.
[42]	104	Extracted human mandibular first molars	Bruker Micro-CT, Kontich, Belgium	12.1 µm	Bruker Micro-CT, Kontich, Belgium	Vertucci’s classification	The mesial RCS were modelled in 3-D and assessed.	The morphology of mandibular molars’ mesial root canals was highly variable. The most common root canal configuration was Vertucci type IV (46.2 percent).	The morphology of the mesial root canals of mandibular molars was discovered to be very diverse in a Brazilian community. Clinicians must have a thorough understanding of the mandibular first molar mesial root canal architecture.
[43]	100	Extracted single-rooted human mandibular incisors	Bruker Micro-CT, Kontich, Belgium	12.1 µm	Bruker Micro-CT, Kontich, Belgium	Vertucci’s classification	A Micro-CT system was used to scan the specimen. At five different levels in the apical third, the software was used to assess the length of the teeth and the number of canal orifices.	The mandibular central and lateral incisors had average lengths of 20.71 and 21.56 mm, respectively. One, two, or three canal orifices were discovered during a cross-section examination of the apical third. According to qualitative assessments of 3-D models of the RCS of the central and lateral incisor teeth, Vertucci’s type I (50 and 62 percent) the most common configuration.	The most common canal configurations in mandibular incisors were Vertucci’s types I and III.
[44]	100	Extracted mandibular first molars	Bruker Micro-CT, Kontich, Belgium	19.6 µm	Bruker Micro-CT, Kontich, Belgium	Vertucci’s classification	The teeth were scanned in a Micro-CT device after being mounted on a custom attachment.	In 76 percent of the distal roots, a single root canal was discovered. In 13%, 8%, and 3% of the sample, respectively, two, three, and four canals were discovered. In 13 cases, the RCS configuration did not fit into Vertucci’s classification.	Single root canals were seen in a significant percentage of the mandibular first molars’ distal roots. Canal configurations not included in Vertucci’s configuration scheme were discovered in 13% of the samples.
[45]	169	Extracted Maxillary first molars	Bruker Micro-CT, Kontich, Belgium	60 µm	Bruker Micro-CT, Kontich, Belgium	Vertucci’s classification	By sectioning the molar at the cementoenamel junction, the palatal root was obtained. Micro-CT was used to scan the roots.	Vertucci type I was used for classifying all canals. Sixty-six percent of the canals had oval cross-sections. In 95 percent of the samples, the major foramen did not line up with the root apex. Straight canals accounted for just 8% of the canals.	Type I was discovered in the palatal roots. However, when treating these roots, several variables must be addressed, such as the frequent occurrence of moderate/severe curvatures, cross-sections, oval-shaped, and the presence of many roots.
[46]	20	Extracted maxillary first molars ***	SkyScan Micro-CT scanner (SkyScan 1172 X-ray Microtomography, Antwerp, Belgium), twelve-bit digital cooled CCD camera with fiber optics	11.6 µm	T Converter (Amira 4.1; Mercury Computer System Inc., Chelmsford, MA, USA) (ECAD-2-12210 PMC; Mercury Computer System Inc.)	Weine and Vertucci’s classification	The mesiobuccal root of the maxillary first molar was placed in a 7 mm plastic container. To remove the adhering from hard and soft tissues, the roots were cleansed, and the mesiobuccal root was removed at the furcation level.	The root canal systems of a large percentage of the roots examined were complex, with 90 percent having a second mesiobuccal canal. Only 60% of root canals could be classified using the Weine classification system, while 70% could be classified using the Vertucci system.	Micro-CT enables a more thorough analysis of root canal anatomy, revealing that the morphology of the mesiobuccal root of maxillary first molar teeth is complicated, and that existing morphological categories are insufficient to capture this complexity.
[47]	340	Extracted Mandibular incisors	Skyscan 1174 (Bruker Micro-CT, Kontich, Belgium)	19 µm	NRecon software, CTAn v.1.12 software (SkyScan, Belgium)	Vertucci classification	The numbers of canals were categorized using the Vertucci classification system, and the apical third was measured in 3-D. For each anatomic categorization, the data was reported as a median and range.	A single root canal was found in all of the specimens (N = 257). Vertucci type III (N = 56) was the second most common morphology. This anatomical group accounts for 92 percent of the total sample. At the 1 mm apical level, oval canals were found 16.7% of the time for Vertucci type I and 37.5 percent of the time for Vertucci type II. At the 3 mm apical level, oval canals increased to 32.4 percent and 76.2 percent for Vertucci type I and III, respectively.	Type I and III forms were found in 92 percent of the mandibular incisors examined. In these morphological configurations, oval-shaped canals in the apical third were common, and they were more common in type III.
[48]	100	Extracted fused-rooted maxillary second molar	Skyscan 1174 (Bruker Micro-CT, Kontich, Belgium)	19.6 µm	(NReconv. 1.6.3; Bruker Micro-CT), CTAn v. 1.16 (Bruker Micro-CT), and CTVolv. 2.3 software (Bruker Micro-CT).	Vertucci classification	At 1, 2, and 3 mm from the anatomical apex of the fused roots, the morphology of the RCS was assessed using the Vertucci classification.	Type 3, Distobucal root fused with Palatal root (27 percent), Type 4, Mesiobucal root fused with Distobucal root, and Palatal root fused with MB or DB roots were the most common root canal fusions (32 percent).	In the root canal system of maxillary second molars with fused roots, merging canals were common.
[49]	118	Mandibular first molars *	Scanco Medical, Bruttisellen, Switzerland	20 µm	Heidelberg, Germany Volume Graphics, VG Studio Max 2.2;	Four-digit system	The pulpal access cavity was prepared with a diamond bur at a high speed, pulp stones were eliminated with an ultrasonic scaler if necessary, and the pulp chamber was rinsed with sodium hypochlorite at the end.	The most common root canal configurations in the mesial root were 2-2-2/2 (31.4%), 2-2-1/1 (15.3%), and 2-2-2/3 (11.9%); there were also 24 additional root canal configurations in this root. In the distal root, 1-1-1/1 (58.5%), 1-1-1/2 (10.2%), and 16 different root canal configurations were found.	The root canal configurations of mandibular first molars vary greatly. Many morphologic differences were found in both the mesial and distal roots.
[50]	123	Maxillary second molar *	Scanco Medical, Bruttisellen, Switzerland	20 µm	Heidelberg, Germany Volume Graphics VG Studio Max 2.2,	Four-digit system	Endodontic access cavities were generated under a microscope for further investigation of the tooth’s internal morphology, making sure not to affect the root canal system morphology or the pulp chamber floor.	The most common root canal configurations in the mesiobuccal root were 2-2-2/2 (19.5%), 2-2-1/1 (14.6%), and 2-1-1/1. (13.0 percent). A total of 93.5 percent of distobuccal roots and 96.7 percent of palatal roots had a 1-1-1/1 arrangement, respectively.	The most common root canal configurations in the mesiobuccal root were 1-1-1/1 (26%).
[51]	116	Maxillary second premolar *	Scanco Medical, Bruttisellen, Switzerland	16 µm	Heidelberg, Germany Volume Graphics VG Studio Max 2.2,	Four-digit system	The maxillary first premolars were scanned with a Micro-CT scanner. The pulp chamber and Root canal configuration (RCC) were depicted in red to identify tooth anatomy, the enamel in white, and the dentin in a transparent grey tint.	The most seen (RCCs) in Maxillary second premolar (Mx2)s) were 1-1-1/1 (35.3%), 1-1-1/2 (21.6%), and 2-1-1/1 (14.7 percent). A total of 11 less common RCCs were discovered. There was just one root in all Mx2Ps.	Within the study’s limits, maxillary second premolars had a significant RCC.
[52]	260	130 Maxillary and 130 Mandibular molars ***	(Bruker micro- CT, Kontich, Belgium) Skyscan 1174	43.3 µm	(Materialize, Leuven, Belgium) Mimics 17.01	Weine’s classification	A semiautomated segmentation method was used to recreate the interior and exterior tooth anatomy. The basic tooth model and the pulp cavity model were combined using a Boolean formula to create a new tooth model. The cavities in this tooth model were filled in. After that, the teeth models were evaluated qualitatively and quantitatively.	A single fused root (51.5 percent) and a single root canal system (49.2 percent) were the most common root/canal types for maxillary molars; typical three-rooted molars were only found in 25.4 percent, and secondary MB canals were only seen in 2 percent. 25% The type 1-1 canal was the most common configuration for mesial and distal root canal systems. A total of 47.7% of mandibular molars were single-rooted, while 32.3 percent had a single root canal system; 20 single-rooted and 60 double-rooted molars had separate mesial and distal root canal systems (61.5%).	The root canal system of the third molars can be physically different in several ways. The degree of canal variation was small in most cases, and the canal form was clear.
[53]	80	Mandibular canines	SkyScan 1173 v2 Micro-CT (Bruker Micro-CT, Kontich, Belgium)	12.1 μm	NRecon software (v1.6.1.0; Bruker, Kontich, Belgium), (CTAn v. 1.14.4, Bruker Micro-CT Kontich, Belgium), and (CTVol v. 2.2.1, Bruker Micro-CT, Kontich, Belgium)	Vertucci’s classification	The number of canals, root canal configurations according to Vertucci’s classification, root length and number and location of lateral canals, the presence of apical delta, perimeter, roundness, and minor and major diameters at cervical, medium, and apical thirds and 1 mm from the foramen were evaluated.	All canals were classified as Vertucci Type I. Lateral canals were verified in 42.4% of the roots, in apical third. The cross-sections at the cementoenamel junction and 1 mm from the apex were oval in 38.3% and 79.4% of the canals, respectively.	The root canal of single-rooted canines evaluated in the present study was classified as Vertucci type I.
[54]	186	Mandibular first premolar	SkyScan 1278, Bruker, Kontich, Belgium	50 μm	CTAn v.1.12 software (Bruker Micro-CT), Kontich, Belgium	Vertucci’s and Ahmed’s classification	All the samples were emerged in 5% sodium hypochlorite for 30 min and reserved in 10% neutral buffered formalin. Dental calculus was removed by using an ultrasonic scaler and stored in a moisturizing solution at room temperature. All teeth were scanned using a high- resolution Micro-CT device.	Radicular grooves were observed in 39.25% of teeth. The ASUDAS scores for radicular grooves were 60.75%, 13.98%, 12.36%, 10.22%, 2.15%, and 0.54%, from grade 0 to grade 5, respectively.	Mandibular first premolars showed a wide range of anatomical variations. Ahmed’s criteria allowed for the classification of the internal anatomy of the root canal in a more precise and practical way than Vertucci’s criteria. Teeth with multiple root canals had a higher incidence of radicular grooves and a more complex morphology compared with teeth with a single root canal.
[55]	520	Mandibular incisors	SkyScan 1176, Bruker Micro-CT, Kontich, Belgium	17.42 μm	CTAn (V1.11.8; SkyScan, Belgium) software, NRecon (V1.6.4,7; SkyScan, Belgium) software, and Data Viewer (V1.5.1.2; SkyScan, Belgium) software	--	All the samples were evaluated 9 mm from the apex using digital radiographs in buccolingual (BL) and mesiodistal (MD) directions. Root canal diameters obtained in measurements were 3, 6, and 9 mm from the apex.	Between all the incisors, 121 (23.3%) were flattened; 215 (41.3%) oval; 142 (27.3%) rounded; 23 (4.5%) round; and 19 (3.6%) with BL flatness 9 mm from the apex.	Oval root canals are predominant in mandibular incisors with a single canal at 9 mm from apex.
[56]	93	Mandibular second molar	Microcomputed tomography (VGStudio Max 2.2; Volume- graphics, Heidelberg, Germany)	20 μm	VGStudio Max 2.2; Volume- graphics, Heidelberg, Germany software	--	Teeth were cleaned and access cavities were prepared. The pulp chamber roof was carefully removed by cutting along the pulp chamber walls. When required, ultrasonic tips were used to re- move pulp stones.	The most frequently observed root canal configurations in the mesial root were 2-2-1/1 (32.3%), 2-2-2/2 (28.0%), 1-1-1/1 (6.5%), and 2-1-1/1 (6.5%); an additional twelve different root canal configurations were also found here. In the distal root, the RCC 1-1-1/1 was observed in 81.7%; another ten different root canal configurations with a frequency of less than 5% were also observed in this root.	The root canal configuration of mandibular second molars showed a great variety. When compared with the first mandibular molar in a historical control from the same sample, the mandibular second molar presented less morphological diversifications.
[57]	47	Maxilary first and second molar	Nikon Metrology Inc, Brighton, MI, USA	--	VG Studio MAX 2.1 software (Volume Graphics GmbH, Heidelberg, Germany)	--	Teeth were mounted on a cylindrical specimen holder and scanned by mCT.	The palatal root of maxillary first molars was found to have statistically significantly thinner dentin than second molars on the palatal aspect of the root 8–11 mm from the apex, correlating to the coronal and middle thirds of the root. First molar palatal roots also had a statistically significantly wider canal mesiodistally than second molars at 13–15 mm from the apex.	The absence of an apical constriction in 76.6% of the specimens highlights the importance of creating an apical seat through instrumentation to maintain obturation materials.
[58]	96	Maxillary first molar misiobuccal root	Micro-CT system (Skyscan 1173; Bruker Co., Kontich, Belgium)	21.39 μm	NRecon soft- ware v.1.6.9.4 (Bruker Co., Kontich, Belgium), InstaRecon^®^ v.1.3.9.2 (IR-CBR Server, University of Illinois Research Park, Illinois, EUA), CTAn v.1.14.4.1, Dataviewer, and CTVox software (Bruker Co., Kontich, Begium)	Weine’s and Vertucci’s classification	Three-Dimensional images of misiobuccal root were analyzed regarding the number of pulp chamber orifices, the number and classification of the canals, the presence of accessory canals in different thirds of the root, and the number and type of apical foramina.	A single entrance orifice was found in 53.0% of the samples, two in 43.9%, and only 3.1% had three orifices. The second mesiobuccal root canal (MB2) was present at some portion of the root in 87.5% of the specimens. A single apical foramen was present in 16.7%, two in 22.9%, and three or more foramina in 60.4% of the roots. Only 55.3% and 76.1% of the root canals could be arranged by Weine’s and Vertucci’s classifications, respectively.	The most commonly found type in this study was Weine type IV/Vertucci type V, and accessory canals were more detected at the apical third, followed by the middle and cervical thirds of the root, respectively.
[59]	374	Mandibular first, second and thirrd molar	Micro-CT scanner (Nanotom S, General Electric)	13.68 μm	CTVox, CTAnalyser and CTVol (SkyScan^®^)	Vertucci’s classification	All the molars were scaned with a Micro-CT scanner (Nanotom S, General Electric)	In the mesial roots of mandibular molars, the most frequent Vertucci type of canal configuration was type IV, except for the mandibular third molar where type I was most common. Type I was most common in the distal root.	Knowledge of the complex anatomy of the mandibular molars can make root canal therapy more likely to succeed.
[60]	89	Mandibular incisor	SkyScan 1173 microtomograph (Bruker Micro–CT Kontich, Belgium)	12.11 μm	NRecon v1.6.6.0 software (Brucker Micro-CT, Kontich, Belgium)	Vertucci’s classification	All the lower incisors were scanned with a micro–CT and reconstructed with NRecon software. Two-Dimensional parameters (perimeter, root length, circularity, and canal diameter) and 3D parameters (volume, surface area, and structure model index) were evaluated with CTAn and CTVol software.	It was found that 89.9% of the canals had a single main root canal (type I), followed by type II (6.7%) and III (3.4%), while 5.6% of the specimens presented lateral canals and 1.1% had an apical delta. Mean volume and surface area were 31.80 mm^3^ and 90.58 mm^2^, respectively. The most prevalent shape of the root canal at CEJ level was circular (41.6%) and 1 mm from the apex, and 73% of the samples were classified as oval.	Incisors have a single root with a relatively simple anatomy, and internal anatomical variations may offer a high degree of technical complexity.
[61]	136	Mandibular first molar	Micro-CT system (mCT-50; Scanco Medical, Bassersdorf, Switzerland)	30 μm	Mimics 18.0 software (Materialise, Leuven, Belgium)	--	All the teeth were scaned with Mimics 18.0 software (Materialise, Leuven, Belgium). The 3-dimensional models of the teeth with root canal systems were constructed and made transparent by adjusting the transparency. The tooth axes based on the shape of the tooth was calculated automatically based on principal component analysis.	The measurements of the maximum curvature of coronal root canals in the axial direction were: in three-canals two-rooted teeth, the average angles of curvatures were 23, 25, and 11 for MB, ML, and DB canals; in four-canals two-rooted teeth were 23, 25, 12, and 16 for MB, ML, DB, and DL canals, respectively; in four-canals three-rooted teeth were 25, 27, 17, and 39 for MB, ML, DB, and DL canals, respectively.	The results of this study are similar to those previously obtained using CBCT and can help us design endodontic approaches.
[62]	30	Premolar	X-Ray microfocus CT scanner (SkyScan 1072; SkyScan, Aart- selaar, Belgium)	19.1 μm	software (NRecon V1.4.0; SkyScan), CT-analyzer V1.6; SkyScan	--	All the radiographs were made in the buccolingual (BL) and mesiodistal (MD) direction to evaluate the root canal anatomy and to identify the radiographic apex using X-ray microfocus CT scanner (SkyScan 1072; SkyScan, Aart- selaar, Belgium).	At all levels of analysis, the BL diameter was greater than the MD diameter for both the canal and the root. Generally, canal and root increased coronally. Buccal and lingual wall thicknesses were greater than mesial and distal at all levels. Canal diameters were at 1 mm from the apex.	Oval canals are frequently present, including in the last few apical millimeters of the root canals.
[63]	105	Mandibular premolar	Micro-CT system (SkyScan 1174v2; Bruker Micro-CT, Kontich, Belgium)	18 μm	NRecon v.1.6.3; Bruker Micro-CT	--	The samples were mounted on a custom attachment and scanned in a Micro-CT system (SkyScan 1174v2; Bruker Micro-CT, Kontich, Belgium)	Overall, specimens had one root with a main canal that divided into mesiobuccal, distobuccal, and lingual canals at the furcation level. Mean length of the teeth was 22.9 and 2.06 mm, and the configuration of the pulp chamber was mostly triangle-shaped. Mean distances from the furcation to the apex and cementoenamel junction were 9.14, 2.07, 5.59, and 2.19 mm.	Type IX configuration of the root canal system was found in 16 of 105 (15.2%) extracted mandibular premolars with radicular grooves.
[64]	72	Maxilary first molar	Micro-CT scanning (SkyScan1174; Bruker Micro-CT, Kontich, Belgium)	22.4 μm	Mimics 15.01 (Materialise, Leuven, Belgium) software	Vertucci’s classification	Each specimen was scanned along the tooth axis with a voxel size of 22.4 μm using Micro-CT scanning (SkyScan1174; Bruker Micro-CT, Kontich, Belgium). The root canal configuration in the MB roots was examined and described by Vertucci’s classification.	MB2 canals were detected in 76.4% (55/72) of the total sample teeth. The incidence of accessory canals was 56.9% (41/72). The mean ratio of D/d was generally “greatest to least”.	The ocrurrence of finding MB2 in maxilary second molar is high.
[65]	178	Mandibular firstpremolar	Micro-CT scanner (Micro-CT Inveon; Siemens Medical Solutions, Knoxville, TN, USA)	15 μm	Cobra software (Siemens Medical Solutions, Knoxville, TN)	Vertucci’s classification	All samples were scanned using a Micro-CT scanner (Micro-CT Inveon; Siemens Medical Solutions, Knoxville, TN) with voxel sizes of 15 μm × 15 μm × 15 μm. The in-built Cobra software (Siemens Medical Solutions, Knoxville, TN) was used for the 3-D reconstruction and analysis.	Almost all the samples were single-rooted (99.4%). In total, 64.04% of teeth possessed type I canal systems, while 34.27% had two canals, and 1.69% had three canals. According to ASUDAS, the scores of radicualr grooves were 56.74%, 16.85%, 12.36%, 10.11%, 3.37%, and 0.56%, respectively, from grade 0 to grade 5. The roots with radicular grooves (grade 3 or 4) were defined as Tome’s anomalous root and these roots have a high incidence of C-shape configurations (66.67%) and multiple-canal systems (100%).	There is obvious variation of the root anatomy and root canal morphology of mandibular first premolar among the southwestern Chinese population, which is very complex and requires careful assessment for endodontic treatment.
[66]	240	Mandibular molar	Micro-CT scanner (SkyScan 1174, SkyScan, Bruker, Belgium)	32.17 μm	software (SkyScan 1174, SkyScan, Bruker, Belgium)	Pomeranz ‘s classification	All the samples were scaned with a Micro-CT scanner (SkyScan 1174, SkyScan, Bruker, Belgium).	The evaluation of three-dimensional (3-D) images of this study showed that no significant difference was found between the percentage of MM (27.5%) and MD canals (22.5%) (*p* = 0.2064); however, there was a significant difference between the percentage of teeth having both extra canals (10%) and teeth having only one of these canals (*p* < 0.05). The confluent configuration (71%) was significantly higher than the other configurations (*p* < 0.05).	The presence of MM canals was higher than that of MD canals; however, the difference was nonsignificant. The occurrence of both extra canals in the same tooth was less significant than the occurrence of only one of either MM or MD canals. The extra canals detected had a higher percentage of the confluent configuration rather than the fin or the independent configurations.
[67]	274	Maxilary pemolars and molar	Micro CT inveon; Siemens Medical Solutions, Knoxville, TN)	15 μm	MICs 10.01 software (Materialise, Leuven, Belgium	Vertucci’s classification	After acess cavity pulp chamber was cleaned and an 15 k file was insered in the canal, X-rays were taken from the misiodital and buccolingual direction.	The root canals of the maxillary posterior teeth showed more significant curvature in the mesiodistal direction than in the buccolingual direction (*p* < 0.05). The MB2 root canal of maxillary molars showed severe bending in the mesiodistal direction: 25.16 ± 6.6 degrees and 28.05 ± 8.65 degrees in first and second molars, respectively. The detection rate of MB2 was 48% in maxillary first molars and 32% in maxillary second molars.	The maxillary posterior teeth showed obvious root canal bending variation and root canal configuration differences. Mostly, the root canals of maxillary premolars showed moderate curvature, while the root canals of maxillary molars showed moderate to severe bending.
[68]	11	Third molar	The Skyscan 1072 X-ray computed microtomograph (Skyscan, Aartselaar, Belgium)	19.74 μm	ANT software (release 2.05, Skyscan, Aartselaar, Belgium)	--	The enamel crown was sealed onto poly (methylmethacrylate) blocks with com- mercial cyanoacrylic glue. Poly (methylmethacrylate) is a radiolucent polymer and these blocks allowed the teeth to be handled easily inside the Micro-CT system. The Skyscan 1072 X-ray computed microtomograph (Skyscan, Aartselaar, Belgium) was used for scanning.	Most roots had a single canal that tapered to the apex. Several canals seemed to have a thin, ribbon-like appearance with focal areas of contact between the walls of the dentine.	Microcomputed tomography seems to be a promising way of studying dental anatomy.
[1]	100	Mandibular canine	lCT scanner (SkyScan 1174v2; Bruker Micro-CT, Kontich, Belgium)	19.6 μm	NRecon v. 1.6.3; Bruker- Micro-CT and CTAn v. 1.12 software (Bruker Micro-CT)	--	After being washed in running water for 24 h, each tooth was dried, mounted on a custom attachment, and scanned in an lCT scanner (SkyScan 1174v2; Bruker Micro-CT, Kontich, Belgium).	The length of the roots ranged from 12.53 to 18.08 mm. Thirty-one specimens had no accessory canals. The location of the apical foramen varied con siderably. The mean distance from the root apex to the major apical foramen was 0.27 and 0.25 mm, and the major diameter of the major apical foramen ranged from 0.16 to 0.72 mm. Mean major and minor diameters of the canal 1 mm short of the foramen were 0.43 and 0.31 mm.	The anatomy and morphology of the root canal of single-rooted canine varied widely in different levels of the root.
[69]	208	Mandibular incisors	Micro-CT scanner (μCT- 50; Scanco Medical, Bassersdorf, Switzerland)	30 μm	MeVisLab v3.2 software (MeVis Medical Solutions AG, Bremen, Germany)	Vertucci’s classification	All the samples were scaned with a Micro-CT scanner (μCT- 50; Scanco Medical).	Three canal categories, labeled as Single (77.88%), Merged (15.87%), and Separated (6.25%), were summarized. The most frequent constriction type in main foramina was single constriction (42.53%). Wide and narrow diameters are in a single main foramen. During the virtual root-end resection, 97.12% of roots underwent successful resection at the 2 mm level.	This study provides detailed information about the root canal morphology and thickness of the crown and root of mandibular incisors in a Chinese population. The most frequent canal configuration was the Single type (77.88%), and more than half (55.2%) of the specimens demonstrated the presence of a constriction.
[70]	101	Maxilary first molar	SkyScan 1272 scanner (Bruker Micro-CT, Belgium)	10 μm	NRecon software (Bruker Micro-CT). CTAn software (Bruker Micro-CT)and CTVol software (Bruker Micro-CT)	Vertucci’s classification	All the samples were scaned with a SkyScan 1272 scanner (Bruker Micro-CT, Belgium).	Eighty-three (82.18%) mesiobuccal roots had multiple canals. The most common canal type is type IV (45.5%), followed by type II (17.8%) and I (17.8%) canals. Type III, V, VI, VII, and VIII canals are less than 10% in total. Seven additional canal types were seen for 10% in total. Fourteen (13.86%) distobuccal roots had multiple canals, and the predominant canal type is type I (86.1%), followed by type II (5.9%) and V (4%) canals. Three additional canal types were observed for 4% in total. All palatal roots possessed the simplest type I canal.	The results of this study reiterate that the root canal configuration of Burmese MFMs is quite complex, especially the mesiobuccal root possessing the highest incidence of additional canals, lateral canals, and apical delta, and isthmuses among three roots.
[71]	100	Maxilary second premolar	SkyScan 1172 X-ray Micro-CT scanner (Bruker Corp., Antwerp, Belgium)	27.4 μm	SkyScan CT-Volume v2.2 software (Bruker Corp., Antwerp, Belgium)	Vertucci classification	All the samples were scaned by SkyScan 1172 X-ray Micro-CT scanner (Bruker Corp., Antwerp, Belgium).	Number of roots were: one root (67%), two roots (30%), three roots (3%), and root canal clasifications were IV and V (both found in 23% of teeth), followed by type I (17%), type III (9%), type II (7%), and type VII (2%).	The root canal morphology of maxillary second premolars in the Saudi Arabian subpopulation is complex and requires cautious evaluation prior to endodontic treatment.
[72]	25	Maxilary second molar	Micro-CT scanner (SkyScan 1174v2; SkyScan N.V., Kontich, Belgium)	22.6 μm	NRecon v1.6.4; SkyScan	--	A Micro-CT scanner (SkyScan 1174v2; SkyScan N.V., Kontich, Belgium) was used for scaning all teeth.	The specimens were classified as types I (*n* = 16), II (*n* = 7), and III (*n* = 2). The size of the roots was similar (*p* > 0.05), and most of them presented straight with one canal, except the mesiobuccal that showed two canals in 24% of the samples.	Considering the evaluation of the external and internal anatomy of four-rooted maxillary second molars, it can be concluded that most of the samples were classified as type I.
[73]	101	Mandibular canine	Bruker SkyScan	10.0 μm	Bruker Micro-CT, Control software version 1.1.19, Kontich, Belgium	--	Bruker SkyScan was ued for scaning for samples.	The root canal configarations were 1-1-1/1 (74.5%) and 1-1-1/2 (14.3%). Physiological foramenwas observed in 80.6% of the MaCas, two in 16.3%, three in 1.0%, and four in 2.0%.	Single-rooted mandibular canines (MaCas) were the most frequently observed (97.0%) ones.
[74]	115	Mandibular first premolar	Siemens Inveon CT, Munich, Germany	14.97 μm	Mimics 10.01 software (Materialise, Leuven, Belgium)	Vertucci classification	Mimics 10.01 software (Materialise, Leuven, Belgium) was used for 3-D imaging.	Canal configuration types I (65.2%), III (2.6%), V (22.6%), and VII were identified (0.9%).	The data obtained in this study revealed com- plex root morphology with a high prevalence of multiple canals, more than half of which exhibited type I canal patterns.
[75]	55	Mandibular first molar	Micro-CT system (SkyScan 1174v2; Bruker- Micro-CT, Kontich, Belgium)	19.6 μm	NRecon v.1.6.3; Bruker Micro-CT, Kontich, Belgium	--	Three-Dimensional models were reconstructed after binarization of the source images, exported by Micro-CT, Kontich, Belgium).	Mesial roots showed a complex distribution of the root canal system in comparison to the distal roots. Almost all distal roots had one root canal and one apical foramen with few accessory canals.	Distolingual roots generally have a short length, severe curvature, and a single root canal with a low apical diameter.
[76]	115	Maxilar first premolar	Micro-CT unit (mCT 40; Scanco Medical, Bru€ttisellen, Switzerland)	16 μm	VGStudio Max 2.2; Volume Graphics, Heidelberg, Germany	--	A Micro-CT unit (mCT 40; Scanco Medical, Bru€ttisellen, Switzerland) was used for scanning all the samples.	Root cannal configarations were in 30 single-rooted teeth, 2-2-2/2 (30.0%), 1-2-2/2 (13.3%), 1-2-1/2 (10%), and 2-2-1/2 (10.0%), and in two-rooted misial roots 1-1-1/1 (56.8%), 1-1-1/2 (29.6%), and 1-1-2/2 (8.6%) in the buccal root, and 1-1-1/1 (92.6%) and 1-1-1/2 (6.2%) in the palatal root’s root canal configaration appeared most frequently.	The results of this study provide detailed morphologic root canal configuration information. Single-rooted teeth showed morphologic diversifications more frequently than two- or three-rooted premolars. Within two-rooted premolars, the buccal root had higher root canal configaration variety, accessory canals, and foramina numbers than the palatal root.
[77]	40	Mandibular first molar	micro- CT scanner (SkyScan 1172 X-ray Micro-CT; SkyScan, Ant- werp, Belgium)	--	SkyScan Micro-CT software for 3-D analysis on sagittal, coronal, and axial slices	Vertucci classification	Forty mandibular first molars were cleaned and stored. Micro-CT scanner (SkyScan 1172 X-ray Micro-CT; SkyScan, Ant- werp, Belgium) were used to scane all the teeth.	The mesial roots of mandibular first molars had canal configurations of type I (15%), type II (7.5%), type III (25%), type IV (10%), type V (2.5%), type VI (7.5%), and type VII (7.5%).	Frequent variations were detected in mesial roots of mandibular first molars. Clinicians should take into consideration the complex structure of the root canal morphology before commencing root canal treatment.
[78]	30	Maxillary first molar	SkyScan 1072, SkyScan b.v.b.a., Aartselaar, Belgium	19.1 μm	NRecon V1.4.0; SkyScan b.v.b.a	Vertucci classification	Maxillary first molar teeth having three separate roots were randomly selected for microtomographic analysis.	The MB2 canal was present in 80% of specimens and was independent in 42% of these cases. When present, the MB2 canal merged with the MB1 canal in 58% of cases.	The MB root canal anatomy was complex: a high incidence of MB2 root canals, isthmuses, accessory canals, apical deltas, and loops was found.
[79]	208	Maxilary first and second molar	CTVox, CTAnalyser and CTVol (SkyScan^®^)	13.68 μm	CTVox, CTAnalyser and CTVol (SkyScan^®^)	Vertucci classification	After cleanig all the maxilary molars, they were scaned by CTVox, CTAnalyser and CTVol (SkyScan^®^).	The mesiobuccal root was the most variable with respect to canal configuration, with type I being the most common configuration followed by type II and type IV. Type I was the most common canal configuration in the distobuccal and palatal root.	It is important to know the morphology of the root canal system in order to perform endodontic treatment correctly.
[22]	50	Mandibular first premolar	Micro-CT scanner (Sky- Scan 1172 X-ray micro-tomograph; SkyScan, Antwerp, Belgium)	11.94 μm	NRecon/InstaRecon reconstruction en- gine; SkyScan	Vertucci’s classification	A Micro-CT scanner (Sky- Scan 1172 X-ray micro-tomograph; SkyScan, Antwerp, Belgium) was used to scan all the mandibular first premolar.	Variable root canal configurations were types I, III, IV, V, and VII. The examined teeth exhibited the following two additional root canal configurations, which did not fit the classification: types 1–2–3 and types 1–3.	A complex morphology of mandibular first premolars were observed with a high prevelence of multiroot canal systems.
[80]	90	Maxilary first molar	HMX 225-ACTIS 4, Tesco, Inc	50 μm	VGStudio Max 2.2; Volume- graphics, Heidelberg, Germany software	Weine’s classification	Before measuring, the 3-D recontracttion was prepared using volumetric analysis.	Single root canal was observed in 44%.	The authors conlcuded that the images were classified based on numeric criteria obatained by Micro-CT.
[81]	46	Maxilary first molar	Micro-CT (SkyScan 1072; SkyScan, Aartselaar, Bel- gium)	19.5 μm	V-Works 4.0; Cybermed, Seoul, Korea	--	Misiobuccal root of maxilary first molar were scaned using Micro-CT (SkyScan 1072; SkyScan, Aartselaar, Belgium).	In these MB roots, 65.2% had two canals, 28.3% had only one canal, and 6.5% had three canals. The most common root canal configuration was two distinct canals (type III: 37.0%), followed by one single canal (type I: 28.3%), two canals that joined together (type II: 17.4%), one canal that split into two (type IV: 10.9%), and three canals (type V: 6.5%).	Micro-CT provided an in-depth analysis of canal configurations, as well as length, curvature, and location of calcified segments.
[12]	78	Maxilary third molar	Micro-CTscanner(SkyScan^®^ 1172, Aartselaar, Belgium)	13.68 μm	NRecon software (SkyScan^®^)	--	All the maxillary third molars were scanned by Micro-CTscanner(SkyScan^®^ 1172, Aartselaar, Belgium).	Maxillary third molars possessed one or three roots, which principally curved buccally/palatally (75.9%), had one to four root canals, and typically no apical constriction (84.4%). The average external root length was 11.89 ± 1.53 mm, while root canal length was 10.18 ± 0.35 mm.	In some cases, the anatomy of maxillary third molars may not be as complicated as previously documented. During root canal treatment, the frequent deviation of the apical foramen from the radiographic apex should be considered, as should the absence of an apical constriction in the majority of cases.
[82]	30	Mandibular first molar	SkyScan 1172; Bruker- Micro-CT, Kontich, Belgium	--	CTVol v. 2.3.2.0 software (Bruker- Micro-CT)	--	All the roots were scaned by SkyScan 1172; Bruker- Micro-CT, Kontich, Belgium.	Mesiobuccal (MB) and mesiolingual (ML) canals were positioned within 2.5 mm from the anatomic apex, and the origin and exit of accessory canals were observed mostly between 1.0 and 2.0 mm from the apex in the group.	The presence of bifid apex in the mesial root of mandibular first molars might be a predictive factor for a complex canal anatomy at the apical third with an increasing number of accessory canals.

* Stored in 70% Alcohol. ** Stored in 0.5% sodium azide solution at 4 °C. *** Stored in 10% formalin.

## Data Availability

The data used in the current study will be made available at a reasonable request.

## Supplementary Materials

The following supporting information can be downloaded at: https://www.mdpi.com/article/10.3390/jcm11092287/s1, Table S1: Quality of included studies by JBI critical appraisal checklist for included studies.

## References

[1] Versiani M.A., Pécora J.D., de Sousa-Neto M.D. (2012). Root and Root Canal Morphology of Four-rooted Maxillary Second Molars: A Micro–Computed Tomography Study. J. Endod..

[2] Bernardi S., Bianchi S., Fantozzi G., Leuter C., Continenza M.A., Macchiarelli G. (2019). Morphometric study on single-root premolars in a European population sample: An update on lengths and diameters. Eur. J. Anat..

[3] Wolf T.G., Kim P., Campus G., Stiebritz M., Siegrist M., Briseño-Marroquín B. (2020). 3-Dimensional Analysis and Systematic Review of Root Canal Morphology and Physiological Foramen Geometry of 109 Mandibular First Premolars by Micro–computed Tomography in a Mixed Swiss-German Population. J. Endod..

[4] Karobari M.I., Noorani T.Y., Halim M.S., Ahmed H.M.A. (2020). Root and canal morphology of the anterior permanent dentition in Malaysian population using two classification systems: A CBCT clinical study. Aust. Endod. J..

[5] Karobari M.I., Noorani T.Y., Halim M.S., Dummer P.M.H., Ahmed H.M.A. (2019). Should inter-canal communications be included in the classification of root canal systems?. Int. Endod. J..

[6] Karobari M.I., Parveen A., Mirza M.B., Makandar S.D., Nik Abdul Ghani N.R., Noorani T.Y., Marya A. (2021). Root and Root Canal Morphology Classification Systems. Int. J. Dent..

[7] Ahmed N., Arshad S., Basheer S.N., Karobari M.I., Marya A., Marya C.M., Taneja P., Messina P., Yean C.Y., Scardina G.A. (2021). Smoking a Dangerous Addiction: A Systematic Review on an Underrated Risk Factor for Oral Diseases. Int. J. Environ. Res. Public Health.

[8] Ahmad I.A. (2015). Root and root canal morphology of Saudi Arabian permanent dentition. Saudi Endod. J..

[9] Sommer L.H. (1957). 0-F. Bennett, PG Campbell and DR Weyenberg. J. Am. Chem. Soc..

[10] Gupta B., Tiwari B., Raj V., Kashyap B., Chandra S., Dwivedi N. (2014). Transparent tooth model: A study of root canal morphology using different reagents. Eur. J. Gen. Dent..

[11] Robertson D., Leeb I.J., Mckee M., Brewer E. (1980). A clearing technique for the study of root canal systems. J. Endod..

[12] Tomaszewska I.M., Leszczyński B., Wróbel A., Gładysz T., Duncan H.F. (2018). A micro-computed tomographic (Micro-CT) analysis of the root canal morphology of maxillary third molar teeth. Ann. Anat.-Anat. Anz..

[13] Grande N.M., Plotino G., Gambarini G., Testarelli L., D’Ambrosio F., Pecci R., Bedini R. (2012). Present and future in the use of Micro-CT scanner 3D analysis for the study of dental and root canal morphology. Annali dell’Istituto Superiore di Sanita.

[14] Acar B., Kamburoğlu K., Tatar İ., Arıkan V., Çelik H.H., Yüksel S., Özen T. (2015). Comparison of micro-computerized tomography and cone-beam computerized tomography in the detection of accessory canals in primary molars. Imaging Sci. Dent..

[15] Solomonov M., Paqué F., Fan B., Eilat Y., Berman L.H. (2012). The Challenge of C-shaped Canal Systems: A Comparative Study of the Self-Adjusting File and ProTaper. J. Endod..

[16] Vertucci F.J. (1984). Root canal anatomy of the human permanent teeth. Oral Surg. Oral Med. Oral Pathol..

[17] Adorno C., Yoshioka T., Suda H. (2010). Incidence of accessory canals in Japanese anterior maxillary teeth following root canal filling ex vivo. Int. Endod. J..

[18] Zillich R., Dowson J. (1973). Root canal morphology of mandibular first and second premolars. Oral Surg. Oral Med. Oral Pathol..

[19] Khedmat S., Assadian H., Saravani A.A. (2010). Root canal morphology of the mandibular first premolars in an Iranian population using cross-sections and radiography. J. Endod..

[20] Awawdeh L., Abdullah H., Al-Qudah A. (2008). Root form and canal morphology of Jordanian maxillary first premolars. J. Endod..

[21] Celikten B., Orhan K., Aksoy U., Tufenkci P., Kalender A., Basmaci F., Dabaj P. (2016). Cone-beam CT evaluation of root canal morphology of maxillary and mandibular premolars in a Turkish Cypriot population. BDJ Open.

[22] Alkaabi W., AlShwaimi E., Farooq I., Goodis H.E., Chogle S.M. (2017). A micro-computed tomography study of the root canal morphology of mandibular first premolars in an Emirati population. Med. Princ. Pract..

[23] Pan J.Y.Y., Parolia A., Chuah S.R., Bhatia S., Mutalik S., Pau A. (2019). Root canal morphology of permanent teeth in a Malaysian subpopulation using cone-beam computed tomography. BMC Oral Health.

[24] Villas-Bôas M.H., Bernardineli N., Cavenago B.C., Marciano M., del Carpio-Perochena A., de Moraes I.G., Duarte M.H., Bramante C.M., Ordinola-Zapata R. (2011). Micro–Computed Tomography Study of the Internal Anatomy of Mesial Root Canals of Mandibular Molars. J. Endod..

[25] Gulabivala K., Aung T., Alavi A., Ng Y.L. (2001). Root and canal morphology of Burmese mandibular molars. Int. Endod. J..

[26] Gulabivala K., Opasanon A., Ng Y.L., Alavi A. (2002). Root and canal morphology of Thai mandibular molars. Int. Endod. J..

[27] Gu Y., Lu Q., Wang H., Ding Y., Wang P., Ni L. (2010). Root Canal Morphology of Permanent Three-rooted Mandibular First Molars—Part I: Pulp Floor and Root Canal System. J. Endod..

[28] Paqué F., Balmer M., Attin T., Peters O.A. (2010). Preparation of Oval-shaped Root Canals in Mandibular Molars Using Nickel-Titanium Rotary Instruments: A Micro-computed Tomography Study. J. Endod..

[29] Guven E.P. (2019). Root Canal Morphology and Anatomy. Human Teeth-Key Skills and Clinical Illustrations.

[30] Hamba H., Nikaido T., Inoue G., Sadr A., Tagami J. (2011). Effects of CPP-ACP with sodium fluoride on inhibition of bovine enamel demineralization: A quantitative assessment using micro-computed tomography. J. Dent..

[31] Huang T.T., Jones A.S., He L.H., Darendeliler M.A., Swain M.V. (2007). Characterisation of enamel white spot lesions using X-ray micro-tomography. J. Dent..

[32] Fan B., Pan Y., Gao Y., Fang F., Wu Q., Gutmann J.L. (2010). Three-dimensional Morphologic Analysis of Isthmuses in the Mesial Roots of Mandibular Molars. J. Endod..

[33] Robinson J.P., Lumley P.J., Claridge E., Cooper P.R., Grover L.M., Williams R.L., Walmsley A.D. (2012). An analytical Micro CT methodology for quantifying inorganic dentine debris following internal tooth preparation. J. Dent..

[34] Rossi-Fedele G., Ahmed H.M.A. (2017). Assessment of root canal filling removal effectiveness using micro–computed tomography: A systematic review. J. Endod..

[35] Joanna Briggs Institute (JBI) (2017). Checklist for Prevalence Studies.

[36] Briseño-Marroquín B., Paqué F., Maier K., Willershausen B., Wolf T.G. (2015). Root canal morphology and configuration of 179 maxillary first molars by means of micro–computed tomography: An ex vivo study. J. Endod..

[37] Wolf T.G., Stiebritz M., Boemke N., Elsayed I., Paqué F., Wierichs R.J., Briseño-Marroquín B. (2020). 3-dimensional Analysis and Literature Review of the Root Canal Morphology and Physiological Foramen Geometry of 125 Mandibular Incisors by Means of Micro–Computed Tomography in a German Population. J. Endod..

[38] Mazzi-Chaves J.F., Silva-Sousa Y.T.C., Leoni G.B., Silva-Sousa A.C., Estrela L., Estrela C., Jacobs R., Sousa-Neto M.D.d. (2020). Micro-computed tomographic assessment of the variability and morphological features of root canal system and their ramifications. J. Appl. Oral Sci..

[39] Domark J.D., Hatton J.F., Benison R.P., Hildebolt C.F. (2013). An ex vivo comparison of digital radiography and cone-beam and micro computed tomography in the detection of the number of canals in the mesiobuccal roots of maxillary molars. J. Endod..

[40] Ordinola-Zapata R., Bramante C., Versiani M., Moldauer B., Topham G., Gutmann J., Nuñez A., Duarte M.H., Abella F. (2017). Comparative accuracy of the Clearing Technique, CBCT and Micro-CT methods in studying the mesial root canal configuration of mandibular first molars. Int. Endod. J..

[41] Kim Y., Chang S.-W., Lee J.-K., Chen I.-P., Kaufman B., Jiang J., Cha B.Y., Zhu Q., Safavi K.E., Kum K.-Y. (2013). A micro-computed tomography study of canal configuration of multiple-canalled mesiobuccal root of maxillary first molar. Clin. Oral Investig..

[42] Marceliano-Alves M.F., Lima C.O., Bastos L.G.d.P.M.N., Bruno A.M.V., Vidaurre F., Coutinho T.M., Fidel S.R., Lopes R.T. (2019). Mandibular mesial root canal morphology using micro-computed tomography in a Brazilian population. Aust. Endod. J..

[43] Leoni G.B., Versiani M.A., Pécora J.D., de Sousa-Neto M.D. (2014). Micro–computed tomographic analysis of the root canal morphology of mandibular incisors. J. Endod..

[44] Filpo-Perez C., Bramante C.M., Villas-Boas M.H., Duarte M.A.H., Versiani M.A., Ordinola-Zapata R. (2015). Micro–computed tomographic analysis of the root canal morphology of the distal root of mandibular first molar. J. Endod..

[45] Marceliano-Alves M., Alves F.R.F., de Melo Mendes D., Provenzano J.C. (2016). Micro–computed tomography analysis of the root canal morphology of palatal roots of maxillary first molars. J. Endod..

[46] Verma P., Love R. (2011). A Micro CT study of the mesiobuccal root canal morphology of the maxillary first molar tooth. Int. Endod. J..

[47] De Almeida M.M., Bernardineli N., Ordinola-Zapata R., Villas-Bôas M.H., Amoroso-Silva P.A., Brandao C.G., Guimaraes B.M., De Moraes I.G., Húngaro-Duarte M.A. (2013). Micro–computed tomography analysis of the root canal anatomy and prevalence of oval canals in mandibular incisors. J. Endod..

[48] Ordinola-Zapata R., Martins J., Bramante C., Villas-Boas M., Duarte M., Versiani M. (2017). Morphological evaluation of maxillary second molars with fused roots: A Micro-CT study. Int. Endod. J..

[49] Wolf T.G., Paqué F., Zeller M., Willershausen B., Briseño-Marroquín B. (2016). Root canal morphology and configuration of 118 mandibular first molars by means of micro–computed tomography: An ex vivo study. J. Endod..

[50] Wolf T.G., Paqué F., Woop A.-C., Willershausen B., Briseño-Marroquín B. (2017). Root canal morphology and configuration of 123 maxillary second molars by means of Micro-CT. Int. J. Oral Sci..

[51] Wolf T.G., Kozaczek C., Campus G., Paqué F., Wierichs R.J. (2020). Root Canal Morphology of 116 Maxillary Second Premolars by Micro–Computed Tomography in a Mixed Swiss-German Population with Systematic Review. J. Endod..

[52] Zhang W., Tang Y., Liu C., Shen Y., Feng X., Gu Y. (2018). Root and root canal variations of the human maxillary and mandibular third molars in a Chinese population: A micro–computed tomographic study. Arch. Oral Biol..

[53] Marceliano-Alves M.F., de Lima C.O., Augusto C.M., Almeida Barbosa A.F., Vieira Bruno A.M., Rosa A.M., Lopes R.T. (2018). The internal root canal morphology of single-rooted mandibular canines revealed by micro-computed tomography. J. Conserv. Dent. JCD.

[54] Sierra-Cristancho A., González-Osuna L., Poblete D., Cafferata E.A., Carvajal P., Lozano C.P., Vernal R. (2021). Micro-tomographic characterization of the root and canal system morphology of mandibular first premolars in a Chilean population. Sci. Rep..

[55] Espir C.G., Nascimento C.A., Guerreiro-Tanomaru J.M., Bonetti-Filho I., Tanomaru-Filho M. (2018). Radiographic and micro-computed tomography classification of root canal morphology and dentin thickness of mandibular incisors. J. Conserv. Dent. JCD.

[56] Wolf T.G., Paqué F., Betz P., Willershausen B., Briseño-Marroquín B. (2017). Micro-CT assessment of internal morphology and root canal configuration of non C-shaped mandibular second molars. Swiss Dent. J..

[57] Divine K.A., McClanahan S.B., Fok A. (2019). Anatomic Analysis of Palatal Roots of Maxillary Molars Using Micro–computed Tomography. J. Endod..

[58] Camargo Dos Santos B., Pedano M.S., Giraldi C.K., De Oliveira J.C.M., Lima I.C.B., Lambrechts P. (2020). Mesiobuccal Root Canal Morphology of Maxillary First Molars in a Brazilian Sub-Population—A Micro-CT Study. Eur. Endod. J..

[59] Tomaszewska I.M., Skinningsrud B., Jarzębska A., Pękala J.R., Tarasiuk J., Iwanaga J. (2018). Internal and external morphology of mandibular molars: An original Micro-CT study and meta-analysis with review of implications for endodontic therapy. Clin. Anat..

[60] Lima C.O., Magalhães L.T., Marceliano-Alves M.F., de Oliveira P.Y., Lacerda M.F. (2020). Internal Lower Incisor Morphology revealed by Computerized Microtomography. Acta Odontol. Latinoam. AOL.

[61] Fu Y., Gao Y., Gao Y., Tan X., Zhang L., Huang D. (2022). Three-dimensional analysis of coronal root canal morphology of 136 permanent mandibular first molars by micro-computed tomography. J. Dent. Sci..

[62] Grande N.M., Plotino G., Pecci R., Bedini R., Pameijer C.H., Somma F. (2008). Micro–computerized tomographic analysis of radicular and canal morphology of premolars with long oval canals. Oral Surg. Oral Med. Oral Pathol. Oral Radiol. Endod..

[63] Ordinola-Zapata R., Bramante C.M., Villas-Boas M.H., Cavenago B.C., Duarte M.H., Versiani M.A. (2013). Morphologic micro-computed tomography analysis of mandibular premolars with three root canals. J. Endod..

[64] Shen Y., Gu Y. (2021). Assessment of the presence of a second mesiobuccal canal in maxillary first molars according to the location of the main mesiobuccal canal-a micro-computed tomographic study. Clin. Oral Investig..

[65] Dou L., Li D., Xu T., Tang Y., Yang D. (2017). Root anatomy and canal morphology of mandibular first premolars in a Chinese population. Sci. Rep..

[66] Alashiry M.K., Zeitoun R., Elashiry M.M. (2020). Prevalence of middle mesial and middle distal canals in mandibular molars in an Egyptian subpopulation using micro-computed tomography. Niger. J. Clin. Pr..

[67] Qiao X., Xu T., Chen L., Yang D. (2021). Analysis of Root Canal Curvature and Root Canal Morphology of Maxillary Posterior Teeth in Guizhou, China. Med. Sci. Monit. Int. Med. J. Exp. Clin. Res..

[68] Guillaume B., Lacoste J.P., Gaborit N., Brossard G., Cruard A., Baslé M.F., Chappard D. (2006). Microcomputed tomography used in the analysis of the morphology of root canals in extracted wisdom teeth. Br. J. Oral Maxillofac. Surg..

[69] Chen M., Wang H., Tsauo C., Huang D., Zhou X., He J., Gao Y. (2022). Micro-computed tomography analysis of root canal morphology and thickness of crown and root of mandibular incisors in Chinese population. Clin. Oral Investig..

[70] Kyaw Moe M.M., Jo H.J., Ha J.H., Kim S.K. (2021). Root Canal Configuration of Burmese (Myanmar) Maxillary First Molar: A Micro-Computed Tomography Study. Int. J. Dent..

[71] Elnour M., Khabeer A., AlShwaimi E. (2016). Evaluation of root canal morphology of maxillary second premolars in a Saudi Arabian sub-population: An in vitro microcomputed tomography study. Saudi Dent. J..

[72] Versiani M.A., Pécora J.D., Sousa-Neto M.D. (2013). Microcomputed tomography analysis of the root canal morphology of single-rooted mandibular canines. Int. Endod. J..

[73] Wolf T.G., Anderegg A.L., Haberthür D., Khoma O.Z., Schumann S., Boemke N., Wierichs R.J., Hlushchuk R. (2021). Internal morphology of 101 mandibular canines of a Swiss-German population by means of Micro-CT: An ex vivo study. Sci. Rep..

[74] Liu N., Li X., Liu N., Ye L., An J., Nie X., Liu L., Deng M. (2013). A micro-computed tomography study of the root canal morphology of the mandibular first premolar in a population from southwestern China. Clin. Oral Investig..

[75] Rodrigues C.T., de Oliveira-Santos C., Bernardineli N., Duarte M.A.H., Bramante C.M., Minotti-Bonfante P.G., Ordinola-Zapata R. (2016). Prevalence and morphometric analysis of three-rooted mandibular first molars in a Brazilian subpopulation. J. Appl. Oral Sci..

[76] Wolf T.G., Kozaczek C., Siegrist M., Betthäuser M., Paqué F., Briseño-Marroquín B. (2020). An Ex Vivo Study of Root Canal System Configuration and Morphology of 115 Maxillary First Premolars. J. Endod..

[77] Şallı G.A., Egil E. (2021). Evaluation of mesial root canal configuration of mandibular first molars using micro-computed tomography. Imaging Sci. Dent..

[78] Somma F., Leoni D., Plotino G., Grande N.M., Plasschaert A. (2009). Root canal morphology of the mesiobuccal root of maxillary first molars: A micro-computed tomographic analysis. Int. Endod. J..

[79] Tomaszewska I.M., Jarzębska A., Skinningsrud B., Pękala P.A., Wroński S., Iwanaga J. (2018). An original Micro-CT study and meta-analysis of the internal and external anatomy of maxillary molars-implications for endodontic treatment. Clin. Anat..

[80] Yamada M., Ide Y., Matsunaga S., Kato H., Nakagawa K. (2011). Three-dimensional analysis of mesiobuccal root canal of Japanese maxillary first molar using Micro-CT. Bull. Tokyo Dent. Coll..

[81] Park J.W., Lee J.K., Ha B.H., Choi J.H., Perinpanayagam H. (2009). Three-dimensional analysis of maxillary first molar mesiobuccal root canal configuration and curvature using micro-computed tomography. Oral Surg. Oral Med. Oral Pathol. Oral Radiol. Endod..

[82] Keleş A., Keskin C., Alqawasmi R., Versiani M.A. (2020). Micro-computed tomographic analysis of the mesial root of mandibular first molars with bifid apex. Arch. Oral Biol..

[83] Versiani M.A., Keleș A. (2020). Applications of Micro-CT technology in endodontics. Micro-Computed Tomography (Micro-CT) in Medicine and Engineering.

[84] Peters O.A., Laib A., Rüegsegger P., Barbakow F. (2000). Three-dimensional analysis of root canal geometry by high-resolution computed tomography. J. Dent. Res..

[85] Ayranci L.B., Yeter K.Y., Arslan H., Kseoğlu M. (2013). Morphology of apical foramen in permanent molars and premolars in a Turkish population. Acta Odontol. Scand.

[86] Guldberg R.E., Lin A.S., Coleman R., Robertson G., Duvall C. (2004). Microcomputed tomography imaging of skeletal development and growth. Birth Defects Res. Part C Embryo Today Rev..

[87] Zhang C., Chen Z., Liu J., Wu M., Yang J., Zhu Y., Lu W.W., Ruan C. (2022). 3D-printed pre-tapped-hole scaffolds facilitate one-step surgery of predictable alveolar bone augmentation and simultaneous dental implantation. Compos. Part B Eng..

[88] Fang H., Zhu D., Yang Q., Chen Y., Zhang C., Gao J., Gao Y. (2022). Emerging zero-dimensional to four-dimensional biomaterials for bone regeneration. J. Nanobiotech..

[89] Swain M.V., Xue J. (2009). State of the art of Micro-CT applications in dental research. Int. J. Oral Sci..

[90] Plotino G., Grande N.M., Pecci R., Bedini R., Pameijer C.H., Somma F. (2006). Three-dimensional imaging using microcomputed tomography for studying tooth macromorphology. J. Am. Dent. Assoc..

[91] Rhodes J., Ford T.P., Lynch J., Liepins P., Curtis R. (1999). Micro-computed tomography: A new tool for experimental endodontology. Int. Endod. J..

[92] Marroquín B.B., El-Sayed M.A., Willershausen-Zönnchen B. (2004). Morphology of the physiological foramen: I. Maxillary and mandibular molars. J. Endod..

[93] Smith T.M., Harvati K., Olejniczak A.J., Reid D.J., Hublin J.J., Panagopoulou E. (2009). Brief communication: Dental development and enamel thickness in the Lakonis Neanderthal molar. Am. J. Phys. Anthropol..

[94] Cheung L.H., Cheung G.S. (2008). Evaluation of a rotary instrumentation method for C-shaped canals with micro-computed tomography. J. Endod..

[95] Luan Q., Desta T., Chehab L., Sanders V., Plattner J., Graves D. (2008). Inhibition of experimental periodontitis by a topical boron-based antimicrobial. J. Dent. Res..

[96] Zhang X., Rahemtulla F., Zhang P., Beck P., Thomas H.F. (2009). Different enamel and dentin mineralization observed in VDR deficient mouse model. Arch. Oral Biol..

[97] Freilich M., Shafer D., Wei M., Kompalli R., Adams D., Kuhn L. (2009). Implant system for guiding a new layer of bone. Computed microtomography and histomorphometric analysis in the rabbit mandible. Clin. Oral Implant. Res..

[98] Anderson P., Yong R., Surman T., Rajion Z., Ranjitkar S. (2014). Application of three-dimensional computed tomography in craniofacial clinical practice and research. Aust. Dent. J..

[99] Loch C., Schwass D.R., Kieser J.A., Fordyce R.E. (2018). Use of micro-computed tomography for dental studies in modern and fossil odontocetes: Potential applications and limitations. NAMMCO Sci. Publ..

[100] Peters O.A., Boessler C., Paqué F. (2010). Root canal preparation with a novel nickel-titanium instrument evaluated with micro-computed tomography: Canal surface preparation over time. J. Endod..

[101] Xu T., Tay F.R., Gutmann J.L., Fan B., Fan W., Huang Z., Sun Q. (2016). Micro–computed tomography assessment of apical accessory canal morphologies. J. Endod..

[102] Baratto Filho F., Zaitter S., Haragushiku G.A., de Campos E.A., Abuabara A., Correr G.M. (2009). Analysis of the Internal Anatomy of Maxillary First Molars by Using Different Methods. J. Endod..

[103] Ahmed H., Hashem A. (2016). Accessory roots and root canals in human anterior teeth: A review and clinical considerations. Int. Endod. J..

[104] Altunsoy M., Ok E., Nur B.G., Aglarci O.S., Gungor E., Colak M. (2014). A cone-beam computed tomography study of the root canal morphology of anterior teeth in a Turkish population. Eur. J. Dent..

[105] Amardeep N.S., Raghu S., Natanasabapathy V. (2014). Root canal morphology of permanent maxillary and mandibular canines in Indian population using cone beam computed tomography. Anat. Res. Int..

[106] Nogueira Leal da Silva E.J., Queiroz de Castro R.W., Nejaim Y., Vespasiano Silva A.I., Haiter-Neto F., Silberman A., Cohenca N. (2016). Evaluation of root canal configuration of maxillary and mandibular anterior teeth using cone beam computed tomography: An in-vivo study. Quintessence Int..

[107] Miyashita M., Kasahara E., Yasuda E., Yamamoto A., Sekizawa T. (1997). Root canal system of the mandibular incisor. J. Endod..

[108] Kartal N., Yanıkoğlu F.Ç. (1992). Root canal morphology of mandibular incisors. J. Endod..

[109] Sert S., Bayirli G.S. (2004). Evaluation of the root canal configurations of the mandibular and maxillary permanent teeth by gender in the Turkish population. J. Endod..

[110] Madeira M.C., Hetem S. (1973). Incidence of bifurcations in mandibular incisors. Oral Surg. Oral Med. Oral Pathol..

[111] Seo M., Park D. (2004). C-shaped root canals of mandibular second molars in a Korean population: Clinical observation and in vitro analysis. Int. Endod. J..

[112] Wang Y., Guo J., Yang H.-B., Han X., Yu Y. (2012). Incidence of C-shaped root canal systems in mandibular second molars in the native Chinese population by analysis of clinical methods. Int. J. Oral Sci..

[113] Zheng Q., Zhang L., Zhou X., Wang Q., Wang Y., Tang L., Song F., Huang D. (2011). C-shaped root canal system in mandibular second molars in a Chinese population evaluated by cone-beam computed tomography. Int. Endod. J..

[114] Fan B., Cheung G.S., Fan M., Gutmann J.L., Bian Z. (2004). C-shaped canal system in mandibular second molars: Part I—anatomical features. J. Endod..

[115] Min Y., Fan B., Cheung G.S., Gutmann J.L., Fan M. (2006). C-shaped canal system in mandibular second molars Part III: The morphology of the pulp chamber floor. J. Endod..

[116] Mohara N.T., Coelho M.S., de Queiroz N.V., Borreau M.L.S., Nishioka M.M., de Jesus Soares A., Frozoni M. (2019). Root anatomy and canal configuration of maxillary molars in a Brazilian subpopulation: A 125-μm cone-beam computed tomographic study. Eur. J. Dent..

[117] Buchanan G.D., Gamieldien M.Y., Tredoux S., Vally Z.I. (2020). Root and canal configurations of maxillary premolars in a South African subpopulation using cone beam computed tomography and two classification systems. J. Oral Sci..

[118] Li Y.-h., Bao S.-j., Yang X.-w., Tian X.-m., Wei B., Zheng Y.-l. (2018). Symmetry of root anatomy and root canal morphology in maxillary premolars analyzed using cone-beam computed tomography. Arch. Oral Biol..

[119] Guo J., Vahidnia A., Sedghizadeh P., Enciso R. (2014). Evaluation of root and canal morphology of maxillary permanent first molars in a North American population by cone-beam computed tomography. J. Endod..

