# Supplementary material for: Root and Root Canal Configuration Characterization Using Microcomputed Tomography: A Systematic Review
Source: J Clin Med. 2022 Apr 20;11(9):2287. doi: 10.3390/jcm11092287 (PMC9099997; doi:10.3390/jcm11092287)
Supplement: Supplementary file 1 [file jcm-11-02287-s001.zip › jcm-1627278-supplementary.pdf]

Table S1. Quality of included studies by JBI critical appraisal checklist for included studies.

Study Name		Question									Overall Scores
		1	2	3	4	5	6	7	8	9	
1	(Briseño-Marroquín et al., 2015)	Yes	Yes	Yes	Yes	Yes	Yes	No	No	Yes	7
2	(Wolf et al., 2020d)	Yes	Yes	Yes	Yes	Yes	Yes	No	No	Yes	7
3	(Wolf et al., 2020a)	Yes	Yes	Yes	Yes	Yes	Yes	No	No	Yes	7
4	(Mazzi-Chaves et al., 2020)	Yes	Yes	Yes	Yes	Yes	Yes	No	Yes	Yes	8
5	(Domark et al., 2013)	Yes	Yes	Yes	Yes	Yes	Yes	No	Yes	Yes	8
6	(Ordinola-Zapata et al., 2017a)	Yes	Yes	Yes	Yes	Yes	Yes	No	Yes	Yes	8
7	(Kim et al., 2013)	Yes	Yes	Yes	Yes	Yes	Yes	No	No	Yes	7
8	(Marceliano-Alves et al., 2019)	Yes	Yes	Yes	Yes	Yes	Yes	No	No	Yes	7
9	(Leoni et al., 2014)	Yes	Yes	Yes	Yes	Yes	Yes	No	Yes	Yes	8
10	(Filpo-Perez et al., 2015)	Yes	Yes	Yes	Yes	Yes	Yes	No	Yes	Yes	8
11	(Marceliano-Alves et al., 2016)	Yes	Yes	Yes	Yes	Yes	Yes	No	No	Yes	7
12	(Verma and Love, 2011)	Yes	Yes	Yes	Yes	Yes	Yes	No	No	Yes	7
13	(De Almeida et al., 2013)	Yes	Yes	Yes	Yes	Yes	Yes	No	No	Yes	7
14	(Ordinola-Zapata et al., 2017b)	Yes	Yes	Yes	Yes	Yes	Yes	No	Yes	Yes	8
15	(Wolf et al., 2016)	Yes	Yes	Yes	Yes	Yes	Yes	No	No	Yes	7
16	(Wolf et al., 2017b)	Yes	Yes	Yes	Yes	Yes	Yes	No	No	Yes	7
17	(Wolf et al., 2020b)	Yes	Yes	Yes	Yes	Yes	Yes	No	No	Yes	7
18	(Zhang et al., 2018)	Yes	Yes	Yes	Yes	Yes	Yes	No	No	Yes	7
19	(Marceliano-Alves et al., 2018)	Yes	Yes	Yes	Yes	Yes	Yes	No	No	Yes	7
20	(Sierra-Cristancho et al., 2021)	Yes	Yes	Yes	Yes	Yes	Yes	No	Yes	Yes	8
21	(Espir et al., 2018)	Yes	Yes	Yes	Yes	Yes	Yes	No	Yes	Yes	8
22	(Wolf et al., 2017a)	Yes	Yes	Yes	Yes	Yes	Yes	No	No	Yes	7
23	(Divine et al., 2019)	Yes	Yes	Yes	Yes	Yes	Yes	No	Yes	Yes	8
24	(Camargo Dos Santos et al., 2020)	Yes	Yes	Yes	Yes	Yes	Yes	No	No	Yes	7
25	(Tomaszewska et al., 2018d)	Yes	Yes	Yes	Yes	Yes	Yes	No	Yes	Yes	8
26	(Lima et al., 2020)	Yes	Yes	Yes	Yes	Yes	Yes	No	No	Yes	7
27	(Fu et al., 2022)	Yes	Yes	Yes	Yes	Yes	Yes	No	Yes	Yes	8
28	(Grande et al., 2008)	Yes	Yes	Yes	Yes	Yes	Yes	No	Yes	Yes	8
29	(Ordinola-Zapata et al., 2013a)	Yes	Yes	Yes	Yes	Yes	Yes	No	Yes	Yes	8
30	(Shen and Gu, 2021)	Yes	Yes	Yes	Yes	Yes	Yes	No	Yes	Yes	8
31	(Dou et al., 2017)	Yes	Yes	Yes	Yes	Yes	Yes	No	No	Yes	7
32	(Alashiry et al., 2020)	Yes	Yes	Yes	Yes	Yes	Yes	No	Yes	Yes	8
33	(Qiao et al., 2021)	Yes	Yes	Yes	Yes	Yes	Yes	No	Yes	Yes	8
34	(Guillaume et al., 2006)	Yes	Yes	Yes	Yes	Yes	Yes	No	No	Yes	7
35	(Versiani et al., 2012a)	Yes	Yes	Yes	Yes	Yes	Yes	No	Yes	Yes	8
36	(Chen et al., 2022)	Yes	Yes	Yes	Yes	Yes	Yes	No	Yes	Yes	8
37	(Kyaw Moe et al., 2021)	Yes	Yes	Yes	Yes	Yes	Yes	No	No	Yes	7
38	(Elnour et al., 2016)	Yes	Yes	Yes	Yes	Yes	Yes	No	No	Yes	7
39	(Versiani et al., 2013)	Yes	Yes	Yes	Yes	Yes	Yes	No	Yes	Yes	8
40	(Wolf et al., 2021)	Yes	Yes	Yes	Yes	Yes	Yes	No	No	Yes	7
41	(Liu et al., 2013b)	Yes	Yes	Yes	Yes	Yes	Yes	No	No	Yes	7
42	(Rodrigues et al., 2016)	Yes	Yes	Yes	Yes	Yes	Yes	No	No	Yes	7
43	(Wolf et al., 2020c)	Yes	Yes	Yes	Yes	Yes	Yes	No	No	Yes	7
44	(Altan Şallı and Egil, 2021)	Yes	Yes	Yes	Yes	Yes	Yes	No	No	Yes	7
45	(Somma et al., 2009)	Yes	Yes	Yes	Yes	Yes	Yes	No	No	Yes	7
46	(Tomaszewska et al., 2018a)	Yes	Yes	Yes	Yes	Yes	Yes	No	Yes	Yes	8
47	(Alkaabi et al., 2017a)	Yes	Yes	Yes	Yes	Yes	Yes	No	Yes	Yes	8
48	(Yamada et al., 2011)	Yes	Yes	Yes	Yes	Yes	Yes	No	No	Yes	7

49	(Park et al., 2009)	Yes	Yes	Yes	Yes	Yes	Yes	No	No	Yes	7
50	(Tomaszewska et al., 2018c)	Yes	Yes	Yes	Yes	Yes	Yes	No	Yes	Yes	8
51	(Keleş et al., 2020)	Yes	Yes	Yes	Yes	Yes	Yes	No	Yes	Yes	8

1. Appropriate sampling frame to address the target population, **2.** Appropriate sampling way of study participants, **3.** Adequate sample size, **4.** Detail description of study participants and settings, **5.** Data analysis with sufficient coverage of the identified sample, **6.** Use of valid methods to identify the condition, **7.** Standard, reliable way of measurement of condition for all participants, **8.** Availability of appropriate statistical analysis, **9.** Adequate response rate and management of low response rate
